# Comparison of weight loss induced by daily caloric restriction versus intermittent fasting (DRIFT) in individuals with obesity: study protocol for a 52-week randomized clinical trial

**DOI:** 10.1186/s13063-022-06523-2

**Published:** 2022-08-29

**Authors:** Danielle M. Ostendorf, Ann E. Caldwell, Adnin Zaman, Zhaoxing Pan, Kristen Bing, Liza T. Wayland, Seth A. Creasy, Daniel H. Bessesen, Paul MacLean, Edward L. Melanson, Victoria A. Catenacci

**Affiliations:** 1 Department of Medicine, Anschutz Health and Wellness Center, University of Colorado Anschutz Medical Campus, Aurora, CO USA; 2 Division of Endocrinology, Metabolism, and Diabetes, Department of Medicine, University of Colorado Anschutz Medical Campus, Aurora, CO USA; 3 Department of Biostatistics and Informatics, University of Colorado Anschutz Medical Campus, Aurora, CO USA; 4 Division of Geriatric Medicine, Department of Medicine, University of Colorado Anschutz Medical Campus, Aurora, CO USA; 5 Eastern Colorado Veterans Affairs Geriatric Research, Education, and Clinical Center, Denver, CO USA

**Keywords:** Weight loss, Calorie restriction, Intermittent fasting, Modified fast, Obesity, Randomized controlled trial

## Abstract

**Background:**

The standard of care for treating overweight and obesity is daily caloric restriction (DCR). While this approach produces modest weight loss, adherence to DCR declines over time and weight regain is common. Intermittent fasting (IMF) is an alternative dietary strategy for reducing energy intake (EI) that involves >60% energy restriction on 2–3 days per week, or on alternate days, with habitual intake on fed days. While numerous studies have evaluated IMF as a weight loss strategy, there are several limitations including lack of a standard-of-care DCR control, failure to provide guideline-based behavioral support, and failure to rigorously evaluate dietary and PA adherence using objective measures. To date, only three longer-term (52-week) trials have evaluated IMF as a weight loss strategy. None of these longer-duration studies reported significant differences between IMF and DCR in changes in weight. However, each of these studies has limitations that prohibit drawing generalizable conclusions about the relative long-term efficacy of IMF vs. DCR for obesity treatment.

**Methods:**

The *D*aily Caloric *R*estriction vs. *I*ntermittent *F*asting *T*rial (DRIFT) is a two-arm, 52-week block randomized (1:1) clinical weight loss trial. The two intervention arms (DCR and IMF) are designed to prescribe an equivalent average weekly energy deficit from baseline weight maintenance energy requirements. Both DCR and IMF will be provided guideline-based behavioral support and a PA prescription. The primary outcome is change in body weight at 52 weeks. Secondary outcomes include changes in body composition (dual-energy x-ray absorptiometry (DXA)), metabolic parameters, total daily energy expenditure (TDEE, doubly labeled water (DLW)), EI (DLW intake-balance method, 7-day diet diaries), and patterns of physical activity (PA, activPAL device).

**Discussion:**

Although DCR leads to modest weight loss success in the short-term, there is wide inter-individual variability in weight loss and poor long-term weight loss maintenance. Evidence-based dietary approaches to energy restriction that are effective long-term are needed to provide a range of evidence-based options to individuals seeking weight loss. The DRIFT study will evaluate the long-term effectiveness of IMF vs. DCR on changes in objectively measured weight, EI, and PA, when these approaches are delivered using guideline-based behavioral support and PA prescriptions.

**Supplementary Information:**

The online version contains supplementary material available at 10.1186/s13063-022-06523-2.

## Administrative information

Note: the numbers in curly brackets in this protocol refer to SPIRIT checklist item numbers. The order of the items has been modified to group similar items (see http://www.equator-network.org/reporting-guidelines/spirit-2013-statement-defining-standard-protocol-items-for-clinical-trials/).

**Table:** 

Title {1}	Comparison of weight loss induced by Daily Caloric Restriction versus Intermittent Fasting (DRIFT) in individuals with obesity: Study protocol for a 52-week randomized clinical trial
Trial registration {2a and 2b}.	ClinicalTrials.gov Identifier NCT03411356. Registered trial name: Intermittent Fasting Versus Daily Caloric Restriction for Weight Loss. Registered on January 26, 2018.
Protocol version {3}	This study protocol was based on version date February 18, 2021.
Funding {4}	This work was supported by grants from the National Institutes of Health: R01 DK111622, P30 DK048520, UL1 TR002535, F32 DK122652, F32 DK123878, K01 HL143039, K01 HL145023. Dr. Melanson is supported by resources from the Geriatric Research, Education, and the Clinical Center at the Denver VA Medical Center. The contents do not represent the views of the U.S. Department of Veterans Affairs or the United States Government
Author details {5a}	^1^ Department of Medicine, Anschutz Health and Wellness Center, University of Colorado Anschutz Medical Campus, Aurora, CO, USA ^2^ Division of Endocrinology, Metabolism, and Diabetes, Department of Medicine, University of Colorado Anschutz Medical Campus, Aurora, CO, USA ^3^ Department of Biostatistics and Informatics, University of Colorado Anschutz Medical Campus, Aurora, CO, USA ^4^ Division of Geriatric Medicine, Department of Medicine, University of Colorado Anschutz Medical Campus, Aurora, CO, USA ^5^ Eastern Colorado Veterans Affairs Geriatric Research, Education, and Clinical Center, Denver, CO, USA
Name and contact information for the trial sponsor {5b}	Department of Health and Human Services, National Institutes of Health (NIH), National Institute of Diabetes and Digestive and Kidney Diseases (NIDDK), healthinfo@niddk.nih.gov, 1-800-860-8747
Role of sponsor {5c}	The sponsors played no role in study design, collection, management, analysis, interpretation of data, writing of the report, or the decision to submit the report for publication.

## Introduction {6a}

Restricting daily calorie intake is considered the standard of care for treating obesity and typically produces modest (5–10%), short-term (26 week) weight loss [1]. However, adherence to daily caloric restriction (DCR) decreases markedly over time [2] and many individuals regain significant weight by one year [3–5]. It has become clear that no single dietary approach will produce weight loss in all individuals [5], and the best dietary approach for a given individual is one they can adhere to over time [2, 6]. Thus, novel dietary interventions are needed to provide a range of evidence-based options to effectively treat obesity.

Intermittent fasting (IMF) is an alternative method of reducing energy intake (EI) that is gaining attention as a strategy for weight loss [7–12]. IMF is defined as >60% energy restriction on 2–3 days per week, or on alternate days [13, 14]. IMF may be an appealing strategy because (1) individuals do not have to count and restrict calories every day and (2) the periodic nature of fasting may mitigate the constant hunger associated with DCR [11]. Our 8-week pilot study comparing IMF to DCR demonstrated IMF was safe and tolerable (with a 93% completion rate at 8 weeks among IMF) and produced similar short-term weight loss (IMF −8.2 kg, DCR −7.1 kg) [15]. Surprisingly, after 6 months of unsupervised follow-up, fat mass continued to decrease in IMF (−0.5±0.8 kg) whereas it increased in DCR (1.2±0.8 kg). In addition, almost twice as many IMF participants (55% vs. 30%) maintained a ≥5-kg weight loss. Thus, IMF may be a more effective dietary strategy for sustained weight loss as compared to DCR; however, more longer-term (≥52 weeks) studies are needed.

While there have been numerous recent studies that have evaluated IMF as a weight loss strategy [16], there are several limitations that prevent a clear understanding of the extent to which IMF is a durable strategy for obesity treatment. These limitations include (1) a lack of a standard-of-care DCR control and (2) not meeting the obesity treatment guidelines regarding either behavioral support or physical activity (PA) recommendations [14]. Current guidelines for obesity treatment recommend a high-intensity (≥14 sessions in 26 weeks), comprehensive behavioral weight loss intervention provided in individual or group sessions by a trained interventionist with principle components of a moderately reduced calorie diet, increased PA, and behavioral support to facilitate adherence for all individuals with BMI ≥ 25 kg/m^2^ [17]. Furthermore, the impact of IMF on PA is largely unexamined. Given the importance of PA for long-term weight loss maintenance [18], the impact of IMF on PA needs to be characterized before IMF is recommended more broadly. Recently, there have been three trials published that have evaluated IMF interventions >26-week duration [19–21]. None of these three longer-duration studies reported significant differences between IMF and DCR in changes in weight. However, each of these studies has limitations that prohibit drawing generalizable conclusions about the relative long-term efficacy of IMF compared to DCR for obesity treatment. These three longer-term studies are described in detail below.

Trepanowski et al. compared the effects of DCR vs. alternate day fasting (ADF) vs. a no-intervention control on weight loss at 52 weeks among 100 adults with overweight or obesity (age 18–64, BMI 25–40 kg/m^2^, 86% female) [19]. The DCR protocol prescribed consuming 75% of weight maintenance requirements in weeks 0–26 and 100% of weight maintenance requirements in weeks 27–52. The ADF protocol prescribed consuming 25% of baseline energy needs on fast days and 125% of baseline EI on alternating “feast” days during the weight loss phase (weeks 0–26) and then 50% of baseline energy needs on fast day and 150% of baseline energy needs on alternating “feast” days during the weight maintenance phase (weeks 27–52). Mean weight loss was similar for participants in DCR and ADF at week 26 (−6.8% in DCR vs. −6.8% in ADF) and week 52 (−5.3% in DCR vs. −6.0% in ADF) relative to those in the control group. The dropout rate was higher in the ADF group (29% in DCR, 38% in ADF, 26% in control), raising concerns about long-term tolerance of ADF [19]. In addition, during the initial 13 weeks of the study intervention, participants in DCR and ADF were not provided any behavioral weight loss support and were provided all meals, limiting real-world applicability. Furthermore, participants were instructed to not change their PA, which is not consistent with current guidelines for obesity treatment [17]. Finally, this study involved only a 26-week weight loss phase, with participants in the ADF group encouraged to eat 150% of energy requirements on fed days during the weight maintenance phase (weeks 27–52), which may have contributed to the higher attrition.

Headland et al. compared weight loss at 52 weeks in 332 adults with overweight or obesity (age 18–72, BMI ≥ 27 kg/m^2^, 83% female) across three randomized groups: DCR (4200 kJ/day for women and 5040 KJ/day for men), a 5:2 IMF protocol (prescribing 2100 kJ/day for women and 2520 kJ/day for men on 2 modified fast days per week), or a week-on-week-off energy restriction prescription (alternating between the DCR energy restriction levels for a week followed by a week of habitual diet) [20]. There were no significant between-group differences in weight loss (−6.6 kg in DCR, −5.0 kg in 5:2 IMF, −5.1 kg in week-on-week-off), body composition, glucose, lipids, or attrition at 52 weeks. However, the overall rate of attrition was high (60% in DCR, 49% in 5:2 IMF, and 58% in week-on-week-off), which limits the generalizability and likely impacted the intent-to-treat results. In addition, the behavioral support was minimal, with participants receiving initial advice from a dietitian, but no ongoing individual or group-based support. Finally, while participants were given a PA goal of 10,000 steps per day, between-group differences in PA levels were not reported.

Lastly, Carter et al. compared weight loss among 137 adults with type 2 diabetes mellitus and overweight or obesity (age ≥ 18, BMI ≥ 27 kg/m^2^, 56% female), randomized to either a DCR protocol (1200–1500 kcal/day) or a 5:2 IMF protocol (2100 kJ/day for women and 2500 kJ/day for men on 2 non-consecutive days/week) for 52 weeks. Participants received individualized behavioral support sessions with a dietician biweekly for the first 3 months and every 2–3 months for the remaining 9 months, with no guidance to increase PA. Attrition was similar across groups (29%) and mean weight change at 52 weeks (−5.0 kg in DCR, −6.8 kg in IMF) and 24 months (−3.9 kg in DCR, −3.9 kg in IMF) was similar between groups [21, 22]. However, this weight loss intervention did not meet the obesity treatment guidelines regarding either behavioral support or PA recommendations. Furthermore, adults with type 2 diabetes mellitus typically have a more difficult time losing weight as compared to adults without diabetes [23], which limits the generalizability of results.

Thus, additional, longer-term randomized trials are needed to evaluate the effectiveness of IMF as compared to DCR on changes in weight, EI, and PA, when these approaches are delivered using guideline-based behavioral support and PA prescriptions. Provision of a comprehensive behavioral support program will likely enhance adherence to IMF and result in greater weight loss and improvement in metabolic outcomes in IMF than observed in prior studies. The *D*aily *C*aloric *R*estriction vs. *I*ntermittent *F*asting Trial (DRIFT) was designed to compare weight loss generated by IMF vs. DCR in adults with overweight or obesity enrolled in a 52-week comprehensive behavioral weight loss intervention. This study will provide translatable clinical data on the effectiveness of a novel dietary weight loss intervention (IMF) as compared to the current standard of care (DCR) over 52 weeks. In addition, this study will use rigorous, objective methods to measure EI and PA, so that adherence to the dietary and PA prescriptions can be compared between IMF and DCR.

### Objectives {7}

The aims of DRIFT are to (1) compare changes in body weight (primary outcome), body composition, and metabolic parameters induced by IMF and DCR; (2) evaluate the impact of IMF vs. DCR on EI and dietary adherence; and (3) evaluate the impact of IMF vs. DCR on energy expenditure (EE) and patterns of PA. Informed by our prior IMF pilot study that suggested that IMF may be better for long-term weight loss maintenance vs. DCR [15], our a priori hypotheses are that IMF will generate greater weight loss (primary outcome), greater decreases in fat mass, improved maintenance of lean mass, and greater improvements in metabolic parameters (fasting lipids, insulin sensitivity, blood pressure) at 52 weeks. While these hypotheses are contrary to recently published data from long-term studies, these data were not available at the time the study was designed. We also will use the National Institutes of Health (NIH) Accumulating Data to Optimally Predict obesity Treatment (ADOPT) framework to evaluate selected biologic, behavioral, environmental, and psychosocial predictors of weight loss response [24–28].

### Trial design {8}

DRIFT is designed as a two-arm randomized controlled trial. Eligible participants will be randomized into one of two study arms: IMF or DCR. This study was powered as a superiority trial. However, if superiority cannot be established, power was also performed for non-inferiority, a priori (see item 14). There are no planned interim analyses. Outcome measures will take place at baseline and weeks, 13, 26, 39, and 52. Selected follow-up measures obtained 26 weeks after completion of the 52-week intervention (i.e., week 78) were added via a protocol amendment after study start. This is not a primary study outcome timepoint, but rather a 26-week post-primary outcome follow-up.

## Methods: participants, interventions, and outcomes

### Study setting {9}

Healthy men and women with overweight or obesity will be recruited from the region around Denver, Colorado. All participants will be recruited and studied at a single site, the University of Colorado Anschutz Medical Campus (CU-AMC).

### Eligibility criteria {10}

Participants will be included in this trial if they are age 18–60 years, have a body mass index (BMI) of 27–46 kg/m^2^, self-report <150 min/week of voluntary exercise at moderate intensity or greater, and <60 min/day of total habitual PA (i.e., work and/or transportation related) at moderate intensity or greater over the past 13 weeks, and if they live or work within 30 min of CU-AMC. Participants will be excluded if they have (1) plans to relocate within the next 52 weeks; (2) plans for extended travel (>2-week continuous travel) within the next 52 weeks; (3) elevated diastolic (>100 mm/Hg) or systolic (>160 mm/Hg) blood pressure; (4) elevated resting heart rate (>100 beats/minute); (5) history of diabetes or fasting glucose ≥126 mg/dL or glycated hemoglobin A1C ≥6.5%); (6) elevated triglycerides (>400 mg/dL); (7) elevated low-density lipoprotein (LDL) cholesterol (>200 mg/dL); (8) untreated thyroid disorder or thyroid stimulating hormone (TSH) level out of the normal reference range; (9) hematocrit, white blood cell count, or platelet count significantly outside the normal reference range; (10) cardiovascular disease, peripheral vascular disease, cerebrovascular disease, significant cardiac arrhythmias, or cardiac valve disease; (11) cancer (within the last 5 years, except skin cancer or other cancers considered cured with excellent prognosis); (12) HIV infection; (13) significant renal, musculoskeletal, neurologic, or hematologic disease; (14) significant gastrointestinal disorders (chronic malabsorptive conditions, peptic ulcer disease, Crohn’s disease, ulcerative colitis, chronic diarrhea, or active gallbladder disease); (15) significant pulmonary disorders (chronic obstructive pulmonary disease , interstitial lung disease, cystic fibrosis, or uncontrolled asthma); (16) regular use of an anti-obesity medication within the last 26 weeks; (17) regular use of prescription or over-the-counter medications known to significantly impact appetite, weight, or energy metabolism within the last 26 weeks (e.g., appetite suppressants, lithium, stimulants, anti-psychotics, tricyclic antidepressants); (18) regular use of systemic steroids (other than oral contraceptive pills) within the last 26 weeks; (19) previous obesity treatment with surgery or weight loss device, except (a) liposuction and/or abdominoplasty if performed >1 year before screening, (b) laparoscopic banding if the band has been removed >1 year before screening, (c) intragastric balloon if the balloon has been removed >1 year before screening, (d) duodenal-jejunal bypass sleeve, if the sleeve has been removed >1 year before screening, or (e) AspireAssist or other endoscopically placed weight loss device if the device has been removed >1 year before screening; (20) current alcohol or substance abuse; (21) current or past (within last 26 weeks) nicotine use; (22) history of clinically diagnosed eating disorders (including anorexia nervosa, bulimia, or binge eating disorder); (23) current severe depression or history of severe depression within the previous year based on DSM-IV-TR criteria for major depressive episode; (24) history of other significant psychiatric illness (e.g., psychosis, schizophrenia, mania, bipolar disorder) which would interfere with the ability to adhere to dietary or exercise interventions; (25) current participation in or plans to participate in any other formal weight loss or PA programs or clinical trials; (26) currently following an intermittent fasting weight loss diet plan; (27) self-reported weight loss >5 kg in past 13 weeks for any reason except post-partum weight loss; (28) self-reported weight loss of >50 lbs. in past 3 years for any reason except post-partum weight loss; (29) urinary incontinence or retention to a degree that would interfere with planned doubly labeled water (DLW) measures; or (30) prescription medication(s) that must be taken with food that would preclude the ability to adhere to the IMF intervention. Females will also be excluded if they are currently pregnant or lactating, were pregnant within the past 26 weeks, are planning to become pregnant in the next 52 weeks, or are sexually active and not using contraception.

### Who will take informed consent? {26a}

Volunteers will be initially assessed for eligibility via a preliminary online screening questionnaire or telephone interview. Potential participants will be asked general eligibility questions regarding age, height, weight, and medical history and receive a brief explanation of the purpose of the study and study expectations, including participant responsibilities and potential risks related to study participation. Interested individuals will be scheduled for a consent and screening visit to determine eligibility. Study coordinators will review the consent form with participants in a private setting. After providing written informed consent, potential participants will undergo a detailed health history and physical examination with the study physician. Body weight and height will be measured to confirm the self-reported BMI. Resting blood pressure and heart rate will be measured, and a resting 12-lead electrocardiogram will be obtained. A fasting venous blood sample will be drawn for measurement of a complete blood count, comprehensive metabolic panel, lipid profile, hemoglobin A1c, and TSH. A urine pregnancy test will be performed in women. Laboratory values available within the past 52 weeks may be substituted for these screening labs at the discretion of the study physician (except for the urine pregnancy test). Participants will also complete the Physical Activity Readiness Questionnaire [29] to assist in screening for exclusionary medical conditions. In addition, participants will complete the Beck Depression Inventory [30] to screen for depression (scores >18 will require further assessment by the study MD to determine if it is appropriate for the subject to participate in the study), the Eating Attitudes Test [31] (scores >20 will require further assessment by the Study MD to determine if it is appropriate for the subject to participate in the study), and the Questionnaire on Eating and Weight Patterns (QEWP-5) [32, 33] to screen for eating disorders (scores that indicate possible diagnosis of a binge eating disorder and/or bulimia nervosa will require further assessment by the Study MD to determine if it is appropriate for the subject to participate in the study). Prior to randomization, all eligible participants will undergo a test fast day to ensure they understand the requirements of the IMF intervention. Participants will be asked to limit EI to 500 kcal/day (women) or 600 kcal/day (men) throughout the test fast day until awakening the following day and rate the difficulty of completing the fast. Study staff will discuss the test fast experience with participants and confirm their willingness to be randomized to the study.

### Additional consent provisions for collection and use of participant data and biological specimens {26b}

During informed consent, participants will be provided an opportunity to consent for additional, optional ancillary studies and storage of data and biological specimens (including blood and stool) to be used in future research, including genetic research.

### Interventions

#### Explanation for the choice of comparators {6b}

We chose to compare IMF to DCR as DCR is the current standard-of-care dietary weight loss intervention. The targeted weekly energy deficit and diet macronutrient content (55% carbohydrates, 15% protein, 30% fat) will be equivalent in the IMF and DCR groups, permitting us to compare the specific effect of the dietary intake pattern on outcomes of interest. Both groups will receive a guideline-based PA prescription [34] and a comprehensive, group-based behavioral weight loss intervention consistent with current guidelines for obesity treatment [17].

### Intervention description {11a}

#### Intermittent fasting (IMF) group

Participants in this group will be instructed to limit EI to 20% of estimated individual daily weight maintenance energy requirements on three non-consecutive days per week. Weight maintenance energy requirements will be calculated as resting energy expenditure (REE) measured by indirect calorimetry multiplied by an activity factor of 1.5 [35]. On fed days, IMF participants will eat ad libitum, but will be encouraged to make healthy food and portion choices. This 80% energy restriction on three fast days per week translates to a targeted weekly energy deficit of ~34%. Participants will be guided to target diet macronutrient content of 55% carbohydrates, 15% protein, and 30% fat. Sample fast day menus and individualized fast day calorie goals will be provided to assist in achieving EI targets. On fast days, participants will be encouraged to consume their calories in their dinner meal. However, they will be allowed to consume their fast day calories at other times of the day if they have difficulty with the recommendation to consume all calories at dinner. A previous study using a similar IMF protocol [36] found that altering times of caloric intake during the modified fast day (i.e., consuming calories at lunch, dinner, or throughout the day) did not impact weight loss or compliance. Participants in this group will be instructed in calorie counting and food logging but will be asked to count calories and log food intake only on fast days.

We chose a modified IMF paradigm rather than a zero-calorie IMF paradigm (i.e., zero-calorie intake on fast days) for several reasons. First, our review of the scientific and lay literature revealed that the strategy largely endorsed is a modified IMF protocol (rather than a zero-calorie IMF protocol); thus, we felt a modified IMF protocol was the most clinically relevant paradigm to study. Second, in a prior study of a zero-calorie IMF intervention, the level of self-reported hunger remained high throughout the study [37]. In contrast, in studies using a modified IMF approach, hunger decreased [38, 39] or remained unchanged [40, 41] compared to pre-intervention levels, suggesting that long-term compliance may be better with a modified IMF protocol. Third, a cross-over study among *n* = 10 adults with overweight or obesity examined food intake 2 days after varying degrees of energy restriction: (1) isoenergetic intake, (2) partial 75% energy restriction (i.e., modified fast day), and (3) 100% energy restriction (i.e., zero-calorie fast day) [42]. Results suggested that a modified fast day produces a similar energy deficit as a zero-calorie fast day over a 3-day period because there is less compensatory increase in EI on the post-fast day with the modified fast [42]. Lastly, we administered a brief online survey to 82 adults with overweight or obesity, who had recently completed a behavioral weight loss interventional trial with similar inclusion and exclusion criteria at our CU-AMC Anschutz Health and Wellness Center; 78% of respondents indicated that they would be more likely to adhere to a modified IMF protocol as compared to a zero-calorie IMF protocol in the context of a 52-week behavioral weight loss program.

#### Daily caloric restriction (DCR) group

Participants in this group will be given a calorie goal designed to produce a 34% energy deficit from baseline estimated individual weight maintenance energy requirements for the duration of the 52-week intervention. As in the IMF group, weight maintenance energy requirements will be determined as (measured REE × activity factor of 1.5) [35]. Participants will be guided to target diet macronutrient content of 55% carbohydrates, 15% protein, and 30% fat.

#### PA prescription

Participants in both groups will also receive a recommendation to gradually increase moderate intensity PA to 300 min/week over the initial 26 weeks and to maintain this level of PA for the duration of the study (see Table 1 for ramp-up). This target is consistent with current PA guidelines for weight management [34, 43]. Participants will be instructed in how to use a relative intensity scale [34] to achieve the target of moderate intensity.

**Table 1:** Description of timeline for PA ramp-up

Study Week	Days/week	Session duration (min/day)	Total duration (min/week)
0	Establish fitness center membership, orientation		
1	3	20	60
2	3	25	75
3–4	3	30	90
5–6	3	35	105
7–8	3	40	120
9–10	3	45	135
11–12	3	50	150
13–14	3	60	180
15–20	4	60	240
21–52	5	60	300

*PA* Physical activity

#### Strategies to improve adherence to interventions {11c}

#### Group-based behavioral support

To improve adherence to the interventions, both groups will receive a 52-week group-based behavioral weight loss program with equivalent contact and support (see Table 2 for a list of weekly behavioral support topics). The programs will fulfill all recommendations for behavioral interventions for treatment of obesity outlined in current guidelines [17]. Randomized groups will meet separately. Curriculum for DCR will be based on the Colorado Weigh behavioral weight loss program which was developed at CU-AMC in 2000 and uses a skills-based approach and cognitive behavioral strategies for lifestyle modification with a dietary focus on DCR [44, 45]. Curriculum for IMF will also be based on the Colorado Weigh behavioral weight loss program and features similar weekly curriculum themes as used in DCR but adapted by the study PI (VC) with input from a registered dietitian (KB) and a behavioral psychologist (AC) to focus on behavioral support specific to IMF. Group meetings will be taught by a registered dietitian with experience in leading group-based behavioral weight loss interventions. Groups will meet weekly during weeks 0–13 and every 2 weeks during weeks 14–52. Weight will be obtained at each group meeting. Group sessions will last ~60 min and the curriculum will be delivered using a mix of large group discussion, small breakout discussions, visual demonstrations, and written exercises. Participants in the IMF group will be instructed in specific strategies to support IMF including strategies to deal with hunger on fast days, distraction techniques, and choosing a balanced diet/appropriate portions on fed days. Participants in the DCR group will be instructed in specific strategies to support DCR with a focus on daily calorie counting and food logging. Topics covered in both groups during the first 26 weeks include realistic weight loss goal setting, self-monitoring strategies, mindful eating, stress management, cognitive restructuring, improving personal food environments and social networks, and strategies to overcome barriers to healthy eating and increasing PA. Later in the curriculum (weeks 27–52), topics will focus on strategies for weight loss maintenance and include impact of weight loss on EE and propensity for weight regain, relapse prevention, and identifying motivation for long-term dietary and PA changes. Participants will also be provided two 10–15 min 1:1 phone calls with their group leader during the initial 26 weeks of the intervention for individualized dietary goal setting using a standardized protocol.

**Table 2:** Weekly behavioral support topics covered in group-based sessions by randomized group

Study week^a^	General behavioral support topic	
	DCR	IMF
0	Introduction to DCR program	Introduction to IMF program
1	Getting started with DCR	Getting started with IMF
2	Portion control	Portion control
3	Food cues and meal planning	Food cues and meal planning
4	Food labels and macronutrient content	Food labels and macronutrient content
5	Wishes vs. reality/self-evaluation 1	Wishes vs. reality/self-evaluation 1
6	Moving those muscles	Moving those muscles
7	Stress management	Stress management
8	Exercise motivation, part 1	Exercise motivation, part 1
9	Dining out	Dining out
10	All about fats	All about fats
11	Mindful eating	Mindful eating
12	Environment	Environment
13	Exercise motivation, part 2	Exercise motivation, part 2
14	Recipe modifications	Recipe modifications
16	Exercise motivation, part 3	Exercise motivation, part 3
18	Cooking demo	Cooking demo
20	Motivation and self-evaluation 2	Motivation and self-evaluation 2
22	Special occasions/holidays	Special occasions/holidays
24	Volumetrics and fiber	Volumetrics and fiber
26	Guest speaker - licensed behavioral psychologist	Guest speaker - licensed behavioral psychologist
28	Boundaries	Boundaries
30	Breaking old habits and creating new ones	Breaking old habits and creating new ones
32	Behavior change identity	Behavior change identity
34	The energy gap	The energy gap
36	Situational and emotional eating	Situational and emotional eating
38	Lapse/relapse/collapse	Lapse/relapse/collapse
40	Fueling for exercise	Fueling for exercise
42	Weight plateaus	Weight plateaus
44	Self-talk	Self-talk
46	National Weight Control Registry	National Weight Control Registry
48	Micronutrients/supplements	Micronutrients/supplements
50	MythBusters	MythBusters
52	The future: ensuring you maintain your weight loss	The future: ensuring you maintain your weight loss

*DCR* daily caloric restriction, *IMF* intermittent fasting

^a^*Note*: These are the general sessions targeted; however, the actual timing of when these sessions will be taught may change based on holiday schedule and class cancelations due to weather

Behavioral support for PA will be provided within the weight loss program and will include discussion of health and weight benefits of PA, weekly PA goals to gradually achieve the target of 300 min/week moderate intensity PA, strategies to overcome barriers to PA, and strategies to improve exercise self-efficacy including exercise goal setting. Participants will also be provided a 20–30-min 1:1 in-person PA support session and a 10–15-min 1:1 follow-up telephone call with an exercise specialist during the initial 26 weeks of the intervention for individualized PA goal setting using a standardized protocol. IMF participants will be guided in strategies to adjust exercise duration on fast days (if needed) and still meet weekly PA targets. Participants will be provided access to the CU-AMC Anschutz Health and Wellness Center Fitness Center for the duration of the intervention.

#### Procedures for monitoring adherence

Adherence to the prescribed dietary interventions for both randomized groups (DCR, IMF) will be monitored via (1) 7-day diet diaries, (2) monthly dietary adherence surveys, and (3) the DLW intake-balance method.

#### Criteria for discontinuing or modifying allocated interventions {11b}

Participants will not be withdrawn for non-adherence in this intent-to-treat study. Participants in both groups will be encouraged to adhere to the dietary prescriptions without modification for the initial 2 weeks as a prior study suggests individuals with obesity become habituated to IMF after ~2 weeks [38]. After the initial 2 weeks, a standardized dietary modification will be offered if a participant (1) expresses a desire to withdraw due to intolerance of study diet and/or (2) experiences adverse effects related to the study diet (i.e., insomnia, impaired concentration, headaches, irritability) that impair ability to function. DCR participants will be allowed to raise their calorie goal to target an energy deficit of 20% from weight maintenance requirements. IMF participants will be allowed to reduce fasting to 2 days per week (i.e., reduce targeted weekly energy deficit to ~20%). Participants will be allowed to continue these strategies for 2 weeks and will then be asked to re-try the original dietary prescription. If they are still unable to tolerate the original dietary prescription after a second attempt, they will be allowed to continue at the modified levels for the study duration. Percentage of participants in each group requiring sustained intervention modification will be recorded. The allocated interventions will not be modified other than as described above. However, participants may discontinue the intervention and/or choose to withdraw from the study at any time.

#### Relevant concomitant care permitted or prohibited during the trial {11d}

Participants will not be permitted to (1) engage in any other formal weight loss or exercise programs or clinical trials during the intervention, (2) use prescription or over-the-counter anti-obesity medications or supplements, and/or (3) undergo obesity treatment with bariatric surgery or endoscopic weight loss device placement.

#### Provisions for post-trial care {30}

Participants will not be provided any compensation if they experience harm or injury from participating in the study. Participants will continue to have access to the printed behavioral weight loss program materials that will be provided during the intervention after completion of study participation.

### Outcomes {12}

Participants will be asked to complete assessment visits at baseline and weeks 13, 26, 39, and 52 during the intervention as well as a follow-up assessment 26 weeks after completion of the 52-week study intervention (i.e., at week 78). See Table 3 for assessments by study timepoint. Body weight, measured in the clinic, is the primary study outcome. Among participants allocated to IMF, outcomes will be measured after a fed day.

**Table 3:**
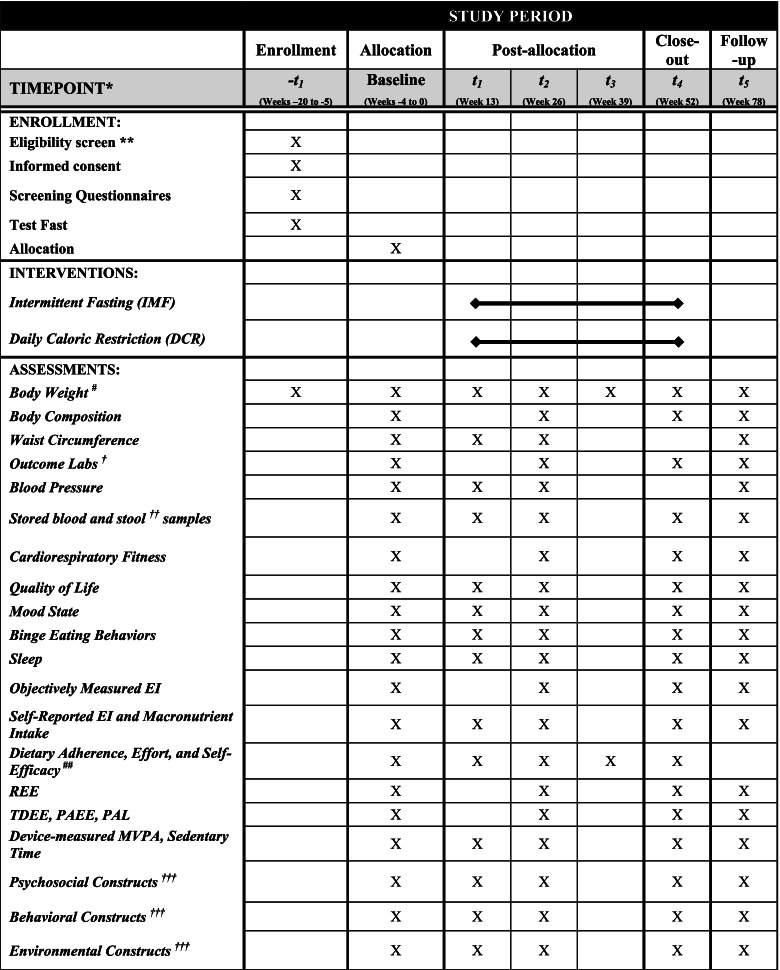
Schedule of enrollment, interventions, and assessments for the DRIFT study participants

*EI* energy intake, *REE* resting energy expenditure, *TDEE* total daily energy expenditure, *PAEE* physical activity energy expenditure, *PAL* physical activity level, *MVPA* moderate-to-vigorous physical activity

*All measures, post-allocation, will occur ±2 weeks from when the measure is due

**Screening measures include blood draw (complete blood count (CBC)), comprehensive metabolic panel (CMP), lipid profile, thyroid stimulating hormone (TSH), and hemoglobin A1C, electrocardiogram, height in centimeters (stadiometer), and physical exam and medical history

^#^Outcome weights will be taken in the morning, after an overnight fast, with the participant wearing a hospital gown. In the IMF group, weight will be taken in the morning following a fed day. Weight will also be measured weekly during weeks 0–26 and every other week during weeks 27–52 at the group-based behavioral weight loss sessions

^##^These measures will be collected monthly

^*†*^Outcome labs include glucose, insulin, triglycerides (TG), free fatty acids (FFA), beta-hydroxybutyrate (BHB), and cortisol. These will be measured at baseline after both a fed (i.e., 12-h fast) and fast day (i.e., 36-h fast, with exception of 25% EI). A lipid panel, insulin, glucose, hemoglobin A1C, leptin, ghrelin, peptide YY, highly sensitive C-reactive protein (hs-CRP), and brain-derived neurotrophic factor (BDNF) will be performed at baseline and weeks 26 and 52

^***††***^ Stored blood and stool samples will be collected at baseline and weeks 13, 26, 52, and 78 in subjects who consented to sample storage

^***†††***^See Table 4 for a detailed list of each construct for psychosocial, behavioral, and environmental factors

#### Anthropometric measures

Body weight will be measured using a digital scale in the CU-AMC Anschutz Health and Wellness Center clinic accurate to ±0.1 kg at baseline and at weeks 13, 26, 39, 52, and 78 in the AM, after an overnight fast. Participants will also be given a smart scale (©BodyTrace smart scales (Palo Alto, CA)) for daily home weighing use during the 52-week study intervention. These daily weights will be transmitted via wireless cellular network to a secure website. Height will be measured to the nearest 1 mm with a stadiometer at baseline. Blood pressure will be measured with a manual sphygmomanometer (average of 2 seated values taken after 5 min rest) at baseline and weeks 13, 26, 52, and 78. Waist circumference (cm) will be measured at baseline and weeks 13, 26, 52, and 78 with a tape measure parallel to the floor, just superior to the iliac crest. Fat mass and lean mass will be measured using dual-energy x-ray absorptiometry (DXA) at baseline and weeks 26, 52, and 78.

#### Resting energy expenditure (REE)

At baseline and weeks 26 and 52, REE will be measured using indirect calorimetry (Parvo Medics TrueOne 2400, Salt Lake City, UT) in the AM in a thermoneutral (68–74 °F) quiet room after a 12-h overnight fast and 24-h abstention from exercise. After the participant has quietly rested for 30 min, a transparent plastic hood connected to the cart will be placed over the participant’s head. For the duration of the test, the participant will be asked to remain motionless and awake. Prior to each measurement, the pneumotach flowmeter will be calibrated using a 3-L calibration syringe (Hans Rudolph Inc., Shawnee, KS, USA), and gas analyzers will be calibrated using standardized gas mixtures. REE will be measured for 20 min and the average of the last 15 min of the measurement will be used to calculate REE using the Weir equation [46].

#### Free-living total daily energy expenditure (TDEE) and energy intake (EI)

TDEE will be measured using the DLW method at baseline and at weeks 26 and 52. Participants will arrive in the AM after a 12-h fast. A baseline urine sample will be collected prior to administration of the oral dose of DLW containing H_2_^18^O (10% atom percent excess, APE) and ^2^H_2_O (99.8% APE) mixed in a ratio of 15:1. Participants will consume a dose based on body weight and an estimation of total body water. Delivered doses will range from 0.85 to 1.10 g/kg of body weight for females and 0.95 to 1.15 g/kg of body weight for males. The dosing cup will be rinsed twice with 30 mL of water and the rinsing dose consumed. The exact time of dosing will be recorded. Urine will be collected after a 4-h and 5-h post-dose equilibrium period and then again on day 8 at the same time points. Sample aliquots (4 mL) will be frozen at −80°C until analysis. Frozen urine samples will be thawed and prepared by centrifugation and analyzed for ^18^O and ^2^H_2_ enrichment by Off-Axis Integrated Cavity Output Spectroscopy (OA-ICOS, Los Gatos Research Inc, Mountain View CA), as previously described [47]. TDEE will be determined using the two-point method according to Speakman et al. [48]; PAEE will be calculated as TDEE (0.9) − REE and PA level (PAL) as TDEE/REE.

EI will be calculated using the intake-balance method. Change in body composition (determined by DXA scan) will be used to calculate change in energy stores (ΔES) using 9.3 and 1.1 kcal/g as the energy coefficients of fat mass and fat free mass respectively [49]. EI will be calculated as TDEE + ΔES/Δtime [50]. However, because DLW cannot quantify macronutrient intake or provide information on patterns of EI on fed and fast days, 7-day diet records will also be collected at baseline and weeks 13, 26, 52, and 78. Diet records will be analyzed by Colorado Clinical and Translational Sciences Institute Nutrition Core personnel blinded to study group assignment using Nutrition Data System for Research software (University of Minnesota).

#### Patterns of PA and sedentary behavior

The activPAL^TM^ micro (activPAL4, PALTechnologies, Glasgow, Scotland) will be used to estimate time spent in moderate-to-vigorous PA (MVPA), light activity, and sedentary behavior over a 7-day period at baseline and weeks 13, 26, 52, and 78. The activPAL4 micro is a small (23.5 × 43 × 5 mm) and light (9.5 g) device that uses accelerometer-derived information about thigh position to estimate time spent in different body positions (i.e., sitting/lying, standing, and stepping). The device is attached to the anterior thigh and is waterproofed by wrapping in a nitrile sleeve. Thus, it can be worn during bathing and overnight, allowing for 24-h measurement of wake and sleep behavior. Raw activPAL.datx files will be processed using PALBatch software using the CREA – 24-h wear protocol (allows for 4 hours of non-wear per day) and auto-correcting for inverted wear (PAL Technologies Ltd., 2019). The algorithm will be used to determine time spent engaging in sedentary behavior, light activity, and MVPA. Many studies have validated the activPAL for use in adults and report very high levels of accuracy (96.2%) for estimating time in activity intensity categories [51–55]. Participants will also be asked to record all exercise and sleep times (i.e., bedtime and waketime) during the 7-day wear period.

#### Self-reported dietary adherence, effort, and self-efficacy

Self-reported dietary adherence and ratings of effort to adhere to the prescribed study diets will be obtained on a monthly basis during weeks 1–52 using methodology described by Dansinger et al. [2]. Participants will be asked to rate on a 1–10 Likert scale: (1) how adherent they were to the prescribed study diet over the past week, (2) how hard was it to adhere to the prescribed study diet over the past week, and (3) how likely they feel they can adhere to the prescribed diet for the next month. Participants in the IMF group will be asked to report the number of fast days over the past week. During weeks 53–78, monthly dietary follow-up questionnaires will be administered to evaluate participant efforts to continue weight loss or weight maintenance after the 52-week intervention has ended.

#### Metabolic/hormonal evaluations

A 12-h fasting blood sample will be obtained for assessment of glucose, insulin, hemoglobin A1c, lipid panel, leptin, ghrelin, peptide YY, and highly sensitive C-reactive protein (hs-CRP) at baseline and weeks 26, 52, and 78. Circulating nutrients and hormonal regulators of fuel metabolism (glucose, insulin, triglycerides, free fatty acids, beta-hydroxybutyrate, and cortisol) will be measured at baseline after both a fed (i.e., 12-h fast) and fast day (i.e., 36-h test fast day with 500 kcal/day (women) or 600 kcal/day (men)) to assess if baseline metabolic response to fasting can serve as a predictor of weight loss. Labs will be analyzed by the UCHealth Clinical Laboratory (beta-hydroxybutyrate) or the Clinical Translational Research Institute (CTRC, all other labs). Insulin sensitivity (homeostasis model assessment of insulin resistance, HOMA-IR) will be calculated as ([insulin] × [fasting glucose × 0.055]/22.5).

#### Psychosocial, behavioral, and environmental predictors of response

To measure psychosocial, behavioral, and environmental constructs which may predict weight loss response, several validated questionnaires and computer-based tasks will be administered to participants. Selection of measures was informed by the recommendations of the NIH ADOPT Core Measures Project [24, 27, 28]. Questionnaires will be administered at baseline and at weeks 13, 26, 52, and 78 (see Table 4). Each month, participants will also be asked to report any changes in their home or work address, to capture changes in their built, social, and community food environments.

**Table 4:** Psychosocial, behavioral, and environmental constructs by study week

		Timepoint (week)				
Questionnaire	Construct measured	0	13	26	52	78
**Psychosocial**						
PANAS [56, 57]	Positive and negative affect	x	x	x	x	x
Perceived Stress Scale (PSS) [58–60]	Perceived stress	x	x	x	x	x
PEMS Coping Subscale [61, 62]	Eating motives	x	x	x	x	x
Binge Eating Scale (BES) [63]	Binge eating	x	x	x	x	x
RED [64]	Reward-based eating drive	x	x	x	x	x
Three-Factor Eating Questionnaire (TFEQ R 18) [65]	Restrained eating, uncontrolled eating, emotional eating	x	x	x	x	x
BREQ-3 [66, 67]	Motivation for exercise	x	x	x	x	x
Grit [68]	Perseverance and passion	x				
TSRQ-Baseline [69]	Motivation to start treatment	x				
TSRQ-Follow-Up [69]	Motivation to continue treatment		x	x	x	x
Mini-IPIP [70]	Personality	x			x	
BARSE [71]	Exercise self-efficacy	x	x	x	x	x
WEL-SF [72]	Diet self-efficacy	x	x	x	x	x
POMS (40-item) [73]	Mood	x	x	x	x	x
RAND-36 [74, 75]	Health-related quality of life	x		x	x	x
Life Events Questionnaire (LEQ) [76, 77]	Stressful life events	x		x	x	x
Behavior-based Identity [78]	Identity	x	x	x	x	x
Role/Group-based Identity Congruence^a^ [79]	Identity congruence	x	x	x	x	x
Emotion Regulation [80]	Emotion regulation	x	x	x	x	x
Psychological Well-Being [81]	Well-being	x	x	x	x	x
Attributional Style [82]	Attribution of events	x	x	x	x	x
Implicit Theory [83]	Beliefs about weight	x	x	x	x	x
Intervention Preference Baseline [study specific]	Intervention preference	x				
Intervention Preference Follow-up [study specific]	Intervention preference		x	x	x	x
Life History Questionnaire [study specific]	Perceived social economic status, developmental history	x^b^				
Computerized task-based assessments	Executive function	x	x	x	x	x
Monetary 5-trial adjusting delay discounting task	Delay discounting	x	x	x	x	x
UPPS+P Questionnaire [84]	Impulsivity	x				
**Behavioral**						
EARLY eating away from home Q [85]	Frequency of eating away from home	x		x	x	x
EARLY SSB Consumption Q [85]	Consumption of sugar-sweetened beverages	x		x	x	x
BRFSS Alcohol Consumption [86]	Alcohol consumption	x		x	x	x
GPAQ, with show cards [87, 88]	Self-reported physical activity	x	x	x	x	x
Munich Chronotype Q (MCTQ) [89]	Sleep	x	x	x	x	x
Marijuana Use (DFAQ-CU) [90]	Marijuana use	x				
BRFSS Marijuana Use [91]	Marijuana use			x	x	x
12-Month Study Questionnaire [study specific]	End of intervention questionnaire				x	
18-Month Study Questionnaire [study specific]	18-month follow-up questionnaire					x
**Environmental**						
NEWS-A [92, 93]	Neighborhood walkability	x				
Social Support for Healthy Behaviors [94]	Perceived social support for healthy eating and PA	x	x	x	x	x

*BARSE* Barriers Self-Efficacy Scale, *BREQ-3* Behavioral Regulations for Exercise Questionnaire version 3, *BRFSS* Behavioral Risk Factor Surveillance System, *DFAQ-CU* Daily Sessions, Frequency, Age of onset and Quantity of Cannabis Use inventory, *EARLY* Early Adult Reduction of weight through LifestYle intervention trials, *GPAQ* Global Physical Activity Questionnaire, *IPIP* International Personality Item Pool, *NEWS-A* Neighborhood Environment Walkability Scale – Abbreviated, *PA *Physical Activity, *PANAS* Positive and Negative Affect Scale, *PEMS* Palatable Eating Motives Scale, *POMS* Profile of Mood States, *Q* Questionnaire, *RED* Reward-based Eating Drive scale, *TSRQ* Treatment Self-Regulation Questionnaire, *UPPS+P* Urgency Premeditation Perseverance Sensation Seeking + Personality Pathway Questionnaire, *WEL-SF* Weight Efficacy Lifestyle Questionnaire Short-Form

^a^Adapted from a measure published by Brook et al. [79]

^b^Optional

Executive control will be assessed using computerized task-based measures of executive function (i.e., attention, inhibition, working memory, and set-shifting) and impulsivity (monetary 5-trial adjusting delay discounting task [95]) performed at baseline and at weeks 13, 26, 52, and 78. The battery of computerized tasks to assess key domains of executive function will be deployed locally on a secure study computer and iPad coded by participant ID. The Urgency Premeditation Perseverance Sensation Seeking + Personality Pathway (UPPS+P) questionnaire [84] will be administered once at baseline as a measure of impulsivity.

### Participant timeline {13}

See Table 3 for the schedule of enrollment, randomization, interventions, and assessments for study participants.

### Sample size {14}

An a priori power calculation was performed for the primary outcome (weight) using nQuery 7.0 (Statistical Solutions Ltd, Cork, Ireland). In our power analyses, we made the following assumptions: (1) power ≥ 0.90 and above for the primary outcome is ideal; (2) the significance level under the null hypothesis will be set at *α* = 0.05; (3) null hypotheses will be tested against two-directional (that is, two-tailed) alternative hypotheses; (4) a 3-kg between-group difference in weight loss was deemed the minimum difference of clinical importance; and (5) the primary analysis will use the intent-to-treat principle in which all randomized participants are analyzed. We will enroll 150 participants (75 participants per randomized group) to provide 90% power at 5% significance to detect a between-group difference of 3 kg in body weight at 52 weeks. With an attrition rate of as high as 20%, we retain 82% power in a sensitivity (completer’s) analysis. Standard deviation (SD) of weight loss used in this calculation was 5.6 kg and was based on a 52-week interventional study using the Colorado Weigh program conducted at our research center [96]. There are no previous data for us to estimate the effect size for any of the outcomes at 52 weeks. If superiority of IMF to DCR cannot be established, we will perform a non-inferiority test using 2.26-kg weight loss as a non-inferiority margin. In this case, we would have 79% power to establish non-inferiority of IMF as compared to DCR assuming no difference in effect of weight loss between the two groups.

### Recruitment {15}

Potential participants will be recruited from the Denver Metro area. We will recruit using CU-AMC campus wide e-mails, flyers, and research website (which maintains an updated list of available studies performed on campus which is also accessible to the general population). We will also provide flyers to the University of Colorado Primary Care practices as well as other local primary care practices including Denver Health Medical Center which serves low-income and minority populations. The trial is registered at ClinicalTrials.gov. We will also employ social media advertising strategies including the use of BUMP Digital Marketing. BUMP uses social media networks (Facebook, Instagram, etc.) to target a population of interest using an appealing advertisement for the study. The advertisement will include a link to a study landing page providing more information about the study, including a brief description of the study requirements and eligibility criteria. Interested individuals will be provided a link to a screening questionnaire.

### Assignment of interventions: allocation

#### Sequence generation {16a}

The randomization assignment will be generated by the study biostatistician (ZP) using a computer-generated block randomization performed within strata defined by sex. Stratification by BMI, age, and race/ethnicity is not needed; given the large sample size, randomization alone should produce balance in these factors.

#### Concealment mechanism {16b}

Allocation of treatment will take place after all eligible participants for each cohort have completed baseline measures, based on the a priori randomization list.

#### Implementation {16c}

The study biostatistician will generate the allocation sequence. After all participants have completed screening and baseline assessments, participants will be notified of the randomization assignment on the first day of group class for the intervention. This process will take place separately within each cohort.

### Assignment of interventions: blinding

#### Who will be blinded {17a}

Due to the nature of the intervention, it is not plausible to blind participants. However, participants will be blinded to the study hypothesis. Due to the nature of the group-based behavioral intervention, it is also not plausible to blind study staff who enroll participants and deliver or monitor intervention delivery. However, the following personnel will be blinded to study group assignment: (1) staff performing laboratory analyses, (2) DLW assessment core staff performing analysis of the DLW samples, and (3) Colorado Clinical and Translational Sciences Institute Nutrition Core personnel who will be using Nutrition Data System for Research software to analyze the 7-day diet diary data. Lastly, all data analyses and reporting will be completed by study investigators who are blinded to study arm assignments.

#### Procedure for unblinding if needed {17b}

Study personnel who are responsible for analyzing data and reporting results will remain blinded throughout the study period.

### Data collection and management

#### Plans for assessment and collection of outcomes {18a}

Outcome measures will be assessed during in-person clinic visits with trained, research staff. We will use detailed data collection forms to promote data quality and reduce assessor error. Weight, blood pressure, and waist circumference will be measured twice and the average of the two measures will be used in analyses. Questionnaires will be administered online using REDCap survey or Qualtrics. Scores from questionnaire responses will be calculated directly within REDCap using hidden, calculated fields. See Table 4 for a list of each study questionnaire and the questionnaire references.

#### Plans to promote participant retention and complete follow-up {18b}

The study coordinators will be responsible for establishing and maintaining contact with participants throughout the study. At enrollment, contact information will be obtained including alternate phone numbers and a phone number of a relative/friend who will always know the participant’s whereabouts. If study appointments are missed, the study coordinator will follow-up with a telephone call to reschedule. If participants miss two group-based support classes in a row without prior communication, study coordinators will contact participants to discuss attendance. In our experience, the most important characteristics of high retention is frequent and high-quality interaction with key study staff. We will ensure continuity of key staff during the entire study so that participants build a relationship with study personnel, to provide inspiration and increase accountability. Participants will also be compensated for completing study outcome measures.

#### Data management {19}

Field and range checks will be programmed to minimize data entry errors. Data distribution will be checked periodically, and outliers verified; missing data will be tracked and checked. All data will be entered into the REDCap database and will be sight verified by a second study staff member.

#### Confidentiality {27}

A participant identifier code for the data will be used so that data will not have the participant’s name associated with it. The key linking participant name and participant identifier code is kept in a secured location, with only key personnel having direct access to the list. Participant identifier codes will be used for all data entry and data analyses. The investigative team will be trained in accordance to both the Colorado Multiple Institutional Review Board (COMIRB) and the Health Insurance Portability and Accountability Act (HIPAA) compliance issues and will act to maintain confidentiality and protect health information. Electronic data will be stored on secure servers with a high level of security, controlled access, daily back-up, and long-term retention of back-up files. Hard copies of study data will be kept in in participants’ charts in a locked, secured file cabinet in the principal investigator’s or study coordinator’s office. Access to all study data will be restricted to the principal investigator and research personnel.

#### Plans for collection, laboratory evaluation, and storage of biological specimens for genetic or molecular analysis in this trial/future use {33}

Blood samples will be collected at baseline and weeks 13, 26, 52, and 78 and will be stored securely until funds are available to support future ancillary studies, including genetic/epigenetic or molecular analyses. Stool samples will be self-collected by study participants at baseline and weeks 13, 26, 52, and 78 using the Alpco EasySampler Stool Collection Kit. Participants will sterilely transfer ~1–2 g of stool to the provided collection tubes, store specimens at −20 °C (home freezers), and transport them to the clinic on icepacks within a week of collection. Blood and stool specimens will then be stored at −80°C for future analyses.

### Statistical methods

#### Statistical methods for primary and secondary outcomes {20a}

Baseline characteristics will be summarized by treatment groups using descriptive statistics. Normality assumptions will be evaluated, and transformations used (e.g., square root and log) as appropriate. Body weight is the primary outcome variable and others are secondary or exploratory outcomes. Consequently, no adjustment for multiple outcomes will be applied. *P*-values < 0.05 will be considered significant. Any imbalance in baseline characteristics between groups that could potentially be a confounding factor for the outcome will be adjusted for in the statistical model.

The primary analysis will use the “intent-to-treat” principle and all randomized participants will be analyzed. Each continuous outcome will be analyzed using a linear mixed effects model. The fixed effects of the linear mixed effects model will consist of treatment group (IMF or DCR) and time of measurement (i.e., baseline and follow-up time points) and their interaction. Time of measurement will be treated as a continuous or categorical variable, as appropriate. Test of the interaction assesses the difference in post-intervention change from baseline between two groups and is used to quantify the effectiveness of the intervention. The 90% confidence interval for the interaction will be calculated. Incidence of adverse events, protocol modifications, and attrition will be compared between groups using chi square or Fisher’s exact test as appropriate.

To explore the impact of selected biologic, psychosocial, behavioral, and/or environmental variables on the intervention effect, we will initially apply models similar to those being tested in aim 1, with the addition of a potential effect moderator as a covariate and its interaction term (i.e., intervention × moderator × time interaction) in addition to related two-way interaction terms. Among factors with a significant three-level interaction, we will further use post-intervention weight loss as the outcome and multiple linear regression to examine their association with the outcome and its interaction with intervention. Similar multiple logistic regression analysis will also be conducted with the outcome dichotomized as responder and non-responder using clinical criteria.

#### Interim analyses {21b}

No interim analyses are planned for this trial.

#### Methods for additional analyses (e.g., subgroup analyses) {20b}

Although not formally powered to examine sex differences, results will be reported separately for men and women to enhance transparency and inform data interpretation.

#### Methods in analysis to handle protocol non-adherence and any statistical methods to handle missing data {20c}

The primary analysis will use the “intent-to-treat” principle to test our study hypotheses under practical and realistic conditions. All randomized participants will be analyzed. We expect that missingness condition is either missing completely at random (MCAR) or missing at random (MAR). A linear mixed effects model will serve as the primary method to handle missing values. The primary and secondary outcomes will be further examined using various sensitivity analyses including analysis of completers using the above statistical models; imputing missing values using baseline-observation-carried-forward, and then assessing efficacy by analyzing the change score from baseline using two sample *t*-tests or linear regression model while adjusting for other covariates; and finally imputing missing values using Markov chain Monte Carlo multiple imputation to create 20 imputed data sets and then analyzing the data using the linear mixed effects model.

#### Plans to give access to the full protocol, participant-level data, and statistical code {31c}

This paper provides the full protocol. Interested individuals should contact the study principal investigator (VC) if interested in other data or documentation of the study.

### Oversight and monitoring

#### Composition of the coordinating center and trial steering committee {5d}

This single-site randomized trial does not have a coordinating center or trial steering committee. The trial will be supervised by the PI (VC) with input from study co-investigators (co-authors EM, DB, PM, and ZP). Additional oversight will be provided by an independent Safety Officer who will review study conduct and progress on an annual basis as outlined in our NIH-approved Data and Safety Monitoring Plan (DSMP). Two senior-level Professional Research Assistants (PRAs, co-authors KB and LW), supervised by the PI, will provide day-to-day organizational support for the trial including participant recruitment and obtaining informed consent and will oversee other study staff (PRAs and/or student workers) in scheduling and performance of outcome measures and data entry. The screening medical history and physical exams will be performed by the PI (VC), co-investigator (DB), and co-author (AZ), all of whom are MDs with current board certification. The PI and the study PRAs will meet weekly to discuss trial conduct and progress including recruitment and retention. Co-investigator meetings will be held monthly during the study startup phase and then as needed to discuss any issues that arise with trial conduct.

#### Composition of the data monitoring committee, its role, and reporting structure {21a}

The intervention and measurement protocols pose minimal risk to participants. Because of this low-risk status, the data safety monitoring plan for this trial focuses on close monitoring by the principal investigator (an MD with current board certification in Endocrinology) in conjunction with an independent Safety Officer, along with prompt reporting of excessive adverse events and any serious adverse events to the NIH and to COMIRB. The Safety Officer will review the reports prepared by the study coordinator at the annual Safety Officer meeting and will determine whether there is any corrective action, trigger of an ad hoc review, or stopping rule violation that should be communicated to the study investigator, COMIRB and the study sponsor.

#### Adverse event reporting and harms {22}

We plan to collect adverse event data on a case-by-case basis. Adverse events will be reviewed, collated, and evaluated by the PI within 72 h. All serious adverse events will be evaluated by the Safety Officer and the PI within 24 h. A summary table of adverse events (Safety Report) will be compiled on an ongoing basis and will be provided to the independent safety officer at the annual meeting. The summary table will include a description of the adverse event, the date of onset, severity, action taken, outcome, whether the adverse event was expected or unanticipated, and a determination of the relationship of the adverse event to study procedures. Adverse event reports and annual summaries will not include identifiable material and participants will be identified only by study ID number.

#### Frequency and plans for auditing trial conduct {23}

To review study conduct throughout the trial period, study team meetings, led by the PI (VC), will occur on a weekly basis. The independent Safety Officer will review trial conduct and progress annually, as outlined in our NIH-approved DSMP. An annual report will be submitted for continuing review per COMIRB requirements. Lastly, an annual progress report will be submitted to the study sponsor (NIH).

#### Plans for communicating important protocol amendments to relevant parties (e.g., trial participants, ethical committees) {25}

Any protocol changes that impact study procedures or risk to human subjects will be approved by COMIRB and reflected in a revised consent form that will be reviewed and signed by all active participants. Protocol changes will also be reported to NIH at the annual progress reports.

#### Dissemination plans {31a}

Findings from this study will be shared publicly and disseminated mainly by publication in peer-reviewed journals and conference presentations. Journal articles will be submitted to PubMed Central in compliance with NIH access guidelines. Main outcomes are planned to be submitted for publication during 2023–2024. Peer-reviewed findings will also be disseminated through CU-AMC social media outlets. Research data, which documents, supports, and validates research findings, will be made available after the main findings from the final research data set have been accepted for publication. No research data with personal identifiers will be published under this project.

## Discussion

Although weight loss interventions based on DCR lead to modest weight loss success in the short-term, there is wide inter-individual variability in weight loss and poor long-term weight loss maintenance. Evidence-based dietary approaches to energy restriction that are effective long-term are needed to provide a range of evidence-based options to individuals seeking weight loss. IMF may represent a promising dietary strategy to help more people achieve weight loss and improve their metabolic health. A recent systematic review identified 27 IMF trials (published between 7/1/2019 and 1/1/2000) reporting weight loss ranging from 0.8 to 13.0% of baseline weight, with no serious adverse events [16]. However, the current evidence base has significant limitations including lack of a standard-of-care DCR control, failure to provide guideline-based behavioral support, and failure to rigorously evaluate dietary and PA adherence using objective measures. To date, only three longer-term (52-week) trials have evaluated IMF as a weight loss strategy [19–21]. None of these longer-duration studies reported significant differences between IMF and DCR in changes in weight. However, each of these studies has limitations that prohibit drawing generalizable conclusions about the relative long-term efficacy of IMF compared to DCR for obesity treatment. Thus, greater scientific rigor is required from interventional trials than found in the current literature and well-designed trials are needed to demonstrate long-term effectiveness and to understand the impact of IMF on energy balance.

DRIFT will fill this gap in the literature by examining the effectiveness of IMF as a dietary strategy to achieve weight loss compared to DCR, the current standard-of-care, within the context of a 52-week, guideline-based behavioral support program for obesity treatment. We will use objective methods to measure EI (DLW intake-balance method) and PA (activPAL device) to allow for an accurate comparison of adherence to the dietary and PA prescriptions between the IMF and DCR groups. This trial is also unique in that it will be one of the first obesity treatment trials to implement the recommendations from the NIH ADOPT framework to evaluate selected biologic, behavioral, environmental, and psychosocial predictors of weight loss response [24–28]. Our findings may have significant public health implications as this study will generate robust data regarding long-term weight loss effectiveness of IMF as compared to DCR. We anticipate that results will further our understanding of the impact of IMF on energy balance and will provide preliminary insight into predictors of weight loss response within IMF and DCR.

## Trial status

This study protocol was based on version date February 18, 2021. Enrollment began on 1/5/2018 and was completed on 4/29/2021. Outcome measures at 78 weeks are expected to be completed by 12/19/2022. We were unable to submit our protocol manuscript prior to completing participant recruitment due to the substantially increased investigator and research staff burden necessitated by COVID-19-related clinical research shutdown activities, followed by COVID-19-related clinical research reactivation activities (including developing, obtaining approval for, and implementation of protocols to safeguard clinical research staff and participants as required by CU-AMC). The additional research burden related to COVID-19 protocols was compounded by frequent staff quarantines and limited access to research offices which required us to focus all investigator and research staff effort on study conduct.

## Supplementary Information

**Additional file 1.** Informed Consent.

## Data Availability

All data that will be used or analyzed in the study will be supplied upon reasonable request.

## Abbreviations

ADF: Alternate day fasting
ADOPT: Accumulating Data to Optimally Predict obesity Treatment
BMI: Body mass index
COMIRB: Colorado Multiple Institutional Review Board
CU-AMC: University of Colorado Anschutz Medical Campus
DCR: Daily caloric restriction
DLW: Doubly labeled water
DRIFT-2: Daily caloric restriction vs. intermittent fasting trial
DSMP: Data and Safety Monitoring Plan
EE: Energy expenditure
EI: Energy intake
ES: Energy stores
HDL: High-density lipoprotein
HIPAA: Health Insurance Portability and Accountability Act
HOMA-IR: Homeostasis model assessment of insulin resistance
hs-CRP: Highly sensitive C-reactive protein
IMF: Intermittent fating
LDL: Low-density lipoprotein
METs: Metabolic equivalents
NIH: National Institutes of Health
PA: Physical activity
PAEE: Physical activity energy expenditure
PAL: Physical activity level
PRA: Professional Research Assistant
QEWP-5: Questionnaire on Eating and Weight Patterns
REE: Resting energy expenditure
RD: Registered dietitian
SD: Standard deviation
TDEE: Total daily energy expenditure
TSH: Thyroid stimulating hormone
