# Supplementary material for: Comparison of weight loss induced by daily caloric restriction versus intermittent fasting (DRIFT) in individuals with obesity: study protocol for a 52-week randomized clinical trial
Source: Trials. 2022 Aug 29;23:718. doi: 10.1186/s13063-022-06523-2 (PMC9421629; doi:10.1186/s13063-022-06523-2)
Supplement: Supplementary file 1 — Additional file 1. Informed Consent. [file 13063_2022_6523_MOESM1_ESM.docx]

**Principal Investigator: Victoria A. Catenacci, MD**

**COMIRB No: 17-0369**

**Version Date: 8.27.2020**

**Study Title: Comparison of Weight Loss Induced by Intermittent Fasting Versus Daily Caloric Restriction in Individuals with Obesity: A 1-Year Randomized Trial**

You are being asked to be in a research study. This form provides you with information about the study. A member of the research team will describe this study to you and answer all of your questions. Please read the information below and ask questions about anything you don’t understand before deciding whether or not to take part.

**Why is this study being done?**

This study plans to learn more about the best eating patterns for weight loss. Specifically, this study is being done to try to determine if intermittent fasting is as effective for weight loss as daily calorie restriction.

In this study, we will be evaluating how your body weight, body composition, laboratory values, energy expenditure, and eating and exercise behaviors change during a 12-month weight loss program. We will also evaluate how your biology, life experiences, thought processes, behaviors, and environment predict how much weight you lose and how well you follow the diet and exercise recommendations in the study.

Some of the blood samples collected during this study will be used for genetic research (research about genes that code for traits that are passed on in families) and epigenetic research (research about how these genes are turned on or off by a person’s environment). This will help us learn more about the impact of a person’s genes on weight loss. It will also help us to learn about whether diet or weight loss impacts which genes are turned on or off. This information could help researchers eventually tailor weight loss programs to a person’s genetic and epigenetic profiles

When your samples are used for this kind of genetic and epigenetic research, the results will not be told to you and will not be put in your health records. Your samples will only be used for research and will not be sold. The research done with your samples may help to develop new products in the future, but there is no plan for you to be paid.

You are being asked to be in this research study because you are interested in losing weight.

**Other people in this study**

Up to 350 people from your area will participate in the study.

**What happens if I join this study?**

If you join the study, you will undergo screening evaluations to see if you are eligible to be in the study. If you are eligible to be in the study, you will be randomized to one of two study groups. Which group you are assigned to will be chosen by chance by a procedure similar to the toss of a coin.

Both study groups will receive weight loss group meetings which focus on changing your eating and activity behaviors for 12 months. Group meetings are taught by a registered dietitian and will occur weekly during months 0-3 and every 2 weeks during months 4-12. Both study groups will reduce their overall calorie intake each week by the same amount (approximately 30%). One group will use intermittent fasting (**IMF**) and the other group will use daily calorie restriction **(DCR**) to reduce their calorie intake. Both groups will also be asked to gradually increase their level of physical activity to a target of 300 minutes of moderate intensity activity per week.

- The **IMF group** will be asked to perform a modified fast on 3 days per week. On the 3 modified fast days they will be asked to eat about 20% of their energy needs (approximately 400-600 calories per day depending on the person). On the 4 other days per week, they will be asked to eat healthy foods and portions, but will not restrict calories those days.
- The **DCR group** will be asked to reduce their calorie intake every day. They will eat about 70% of their energy needs every day.

**Your participation in the study will last approximately 18 months.** You will receive a 12 month weight loss intervention and undergo periodic assessments of your body weight, body composition, laboratory values, energy expenditure, and eating and exercise behaviors. These are described in more detail in section 2 below.

During the 18 months you are enrolled in the study, you will be asked not to participate in any other weight loss or exercise programs. You will also be asked not to participate in any other research studies involving weight loss or exercise. You will be asked not to take any weight loss medications or supplements. If you are female and can become pregnant, you will be asked not to become pregnant and to use a reliable method of birth control.

If you join the study, you will be asked to undergo the following steps:

1. **Screening Procedures**

You will be asked to have the following screening procedures done to see if you are eligible to be in this study. These screening procedures will take about 3-4 hours and will be divided over 2-3 visits. The procedures will be performed on the Anschutz Medical Campus of the University of Colorado at either the Anschutz Health and Wellness Center (AHWC) or the Clinical Translational Research Center (CTRC) outpatient clinic. The address of the AHWC is 12348 E. Montview Boulevard, Aurora, CO 80045. The outpatient CTRC is located on the third floor of the Leprino Building, 12401 E. 17th Avenue, Aurora CO 80045.

a. Review of the Consent Form. This consent form will be reviewed with you in detail and you will initial each page and sign the last page if you agree to be in the study. No other procedures will occur until you sign the consent form.

b. Vital Signs and Body Measurements. Your blood pressure and heart rate will be measured. Your height and weight will be measured.

c. Health and Physical Exam. A standard medical history will be taken and a physical exam will be performed by the study physician.

d. ECG: We will measure your heart rate and rhythm with an electrocardiogram (ECG).

e. Screening Questionnaires. You will be asked to fill out questionnaires that give us information on your demographic information, weight history, medical history, social history, mood, and eating attitudes.

f. Blood Test. You will be asked to have your blood drawn for routine blood tests to further evaluate if you are eligible to be in this study. Approximately 5 teaspoons of blood will be removed by putting a needle into your vein. You may be asked to fast overnight for this test.

g. Pregnancy Test. If you are a female of reproductive age, you will be given a urine pregnancy test. You cannot be in this study if you are pregnant.

h. Test Fast. You will be asked to fast for 36 hours starting at midnight prior to the fast day through approximately 8 AM of the morning following the fast day, except for a light dinner on the fast day (we will provide you with a sample meal menu and a calorie goal). The test fast is designed to be similar to a fast day in the intermittent fasting group. You will be asked to record all food intake during this fast. You will have your blood drawn in the morning of the fast day and again on the morning following the fast day. You will also discuss your experience with study staff either in person or over the telephone.

**2. Randomization.** If you complete the screening procedures and are eligible for the study, you will be randomized to one of two study groups. Which group you are assigned to will be chosen by chance by a procedure similar to the toss of a coin. You will have a 50% chance of being assigned to either group. Both groups will receive a 12-month group-based behavioral weight loss program. Weight loss group meetings will focus on changing your diet and eating behaviors. The difference between the two study groups is that one group will use intermittent fasting (IMF) to reduce calorie intake lose weight and the other group will use daily calorie restriction (DCR) to reduce calorie intake and lose weight. **Randomized groups will meet separately.**

**3. Study Interventions.** Once you are randomized to the study, you will be asked to

undergo the following interventions for research purposes:

1. Weight Loss Group Meetings: You will be asked to attend weight loss group

meetings at the AHWC with about 10-15 other people in the study. Randomized groups will meet separately. Each group will meet on a regular schedule (weekly during months 0-3 and then every two weeks during months 4-12). Meetings will last about 60-90 minutes. Group meetings will be taught by a registered dietitian and will focus on making changes in your diet and eating behaviors to help you lose weight. There will be a mix of lecture presentations, individual worksheets, and discussion during the group meetings. Participants will also have two 1:1 phone calls with their group leader. You will be asked to keep a food log to help you monitor your food intake. You will be weighed at each group meeting. Group meeting attendance and completion of food logs will be tracked to monitor your progress in the weight loss program.

b. Dietary Intervention: Both groups will reduce their overall calorie intake each week by the same amount (approximately 30%) for the duration of the 12-month weight loss intervention. However study groups will use different eating patterns to reduce their weekly calorie intake.

- **If you are randomized to the IMF group, you will be asked to perform a modified fast (eat only 20% of your energy needs) three days a week**. You will be provided a calorie goal and sample menus to help you meet this modified fast day calorie goal. For most people, the calorie goal will be between 400-600 calories a day on the modified fast days. On non-fast days, you will be asked to eat a sensible, healthy diet but you will not have to restrict your calorie intake.
- **If you are randomized to the DCR group you will be asked to eat about 70% of your energy needs every day for the duration of the study**. You will be given a daily calorie goal and sample menus to help you meet this goal.

1. Physical Activity Intervention. You will be asked to gradually increase moderate intensity physical activity (PA) to 300 minutes per week over the initial 26 weeks and to maintain this level of PA during weeks 27-52. Behavioral support for PA will be provided within the group weight loss meetings. In addition, you will have one in-person meeting and a 1:1 phone call with an exercise specialist.

**4. Study Assessments**

a. Body Measurements. Your weight will be measured at the AHWC or CTRC at baseline and weeks 4, 13, 26, 39, 52, and 78 in the morning after you change into a hospital gown. You may also be issued a digital “smart” scale for the 12 month duration of the study weight loss intervention and for the 6 month follow up period and asked to weigh yourself at home daily. These daily weights will be transmitted via wireless cellular network to a secure website. Weight will also be measured at each group weight loss meeting, but you will not have to change into a hospital gown. Your waist circumference will be measured at baseline and weeks 13, 26, 52, and 78. Your blood pressure will be measured at baseline and weeks 13, 26, 52, and 78.

b. Total Daily Energy Expenditure (TDEE). TDEE (the number of calories you burn) will be measured over 7 days using the Doubly Labeled Water method. This method is based on measuring the rates of how you metabolize two unusual forms of water. The unusual forms of water are stable (non-radioactive) isotopes that are safe for human consumption. You will be asked to provide a urine specimen and your resting energy expenditure (REE) may be measured (see below) after an overnight (12 hour) fast on day 1 of the measure. After this you will be given a drink of water containing the two stable isotopes. This special water tastes like regular tap water. After drinking this water you will need to stay in a resting state. During this time you will not be able to eat or drink any food and will be asked not to urinate for 4 hours. You will be asked to provide 2 more urine specimens and 1 saliva sample on day 1. You may also be asked to collect samples of your urine periodically during the week of the doubly labeled water measure. You will need to provide 2 additional urine samples 7 days later. Your REE may be measured (see below) on this day also. This measure of TDEE is performed at baseline and at approximately weeks 26 and 52 in all subjects. About half of the subjects in the study will also be asked do this measure of TDEE at approximately weeks 4 and 78. Thus, you will undergo a total of 3-5 TDEE measurements.

c. Resting Energy Expenditure (REE). REE will be measured using indirect calorimetry. Indirect calorimetry measures the amount of oxygen you use and the amount of carbon dioxide you produce. From these measurements, the number of calories your body uses at rest can be determined. For this test, you will be asked to come to the AHWC or the CTRC in the morning after an overnight (12 hour) complete fast (no food). You will lie quietly for about 30-40 minutes after which a ventilated clear Plexiglas hood will be placed over your head that allows us to collect the air you breathe out and analyze it for oxygen and carbon dioxide. This will take about 15-20 minutes, so the entire procedure will take about 1 hour. You will be required not to engage in any strenuous physical activity for 24 hours prior to the test. These REE measures are performed at baseline and at approximately weeks 26 and 52 in all subjects. About half of the subjects in the study will also be asked do this measure of REE at approximately week 4. Some subjects may also be asked to undergo an REE measurement at week 78. REE may be measured twice at each time point, approximately 1 week apart. Thus, you will undergo a total of 3-10 REE measurements.

d. DXA Scan. You will be asked to come to either the AHWC or the CTRC to measure your level of body fat using a special x-ray. During this procedure, you will lie on a hospital bed and a small amount of x-ray will be passed through your body. This test takes approximately 45 minutes. There is no pain associated with this test. A pregnancy test will be given prior to the scan in all women of child bearing age who have not had their uterus removed. All subjects will have one DXA scan performed at each assessment time point (baseline, and at approximately weeks 26, 52 and 78). Some subjects will be asked to undergo a DXA scan at approximately week 4 and/or a second DXA scan at baseline, week 26 or 52. Thus, you will undergo a total of 4-8 DXA scans.

e. Outcome Questionnaires**:**  You will be asked to complete questionnaires that give us information on your behaviors, mood, feelings, thought processes, and environment. In cohorts impacted by the COVID-19 pandemic, we will also ask you questions about the impact of COVID 19 on your study participation and weight loss efforts. This panel of approximately 25 short surveys will take approximately 1 hour to complete. These surveys can be completed during study visits or we can send you a secure link to complete these questionnaires from you home computer. A few of these questionnaires ask about things that may make you uncomfortable such as how much money you make, alcohol or drug use, and puberty/sexual development. All of your answers will be kept confidential and you do not have to answer any questions that you do not want to answer. These questionnaires will be performed at baseline and at weeks 13, 26, 52 and 78.

f. Executive Function Measures: You will be asked to complete some tasks on a computer to assess your executive function (attention and memory). These measures will take approximately 1 hour to complete, and will be done during the study visits. These measures will be performed at baseline and at weeks 13, 26, 52 and 78.

g. Dietary Record. You will be asked to complete a record in which you will record all of your food and beverage intake for 7 days. These records will be performed at baseline and at approximately weeks 13, 26, 52, and 78.

h. Activity Measurements. You will be asked to wear the ActivPAL physical activity monitor for a 7-day period. The ActivPAL is a small monitor that senses movement and body position that you wear on your thigh. This device is small and will not restrict your movement. You will wear this device all the time for seven days in a row. This week you wear the monitor should be a typical week, for example you should not be going on vacation or performing activities that are unusual for you. You will be asked to log all exercise you perform during the week you wear the activity monitor. You will be asked to return the activity monitor to the investigators promptly after the 7 day period. These activity measurements will be performed at baseline and at weeks 26 and 52. Some subjects will be asked to perform this activity measurement at week 13 and 78.

i. Blood Tests. You will be asked to fast overnight (12 hours) and your blood will be drawn in the morning. The blood tests are done at the outpatient CTRC or the AHWC. Up to 8 teaspoons of blood will be removed by putting a needle into your vein. Your blood will be drawn in the morning after an overnight fast on 2 consecutive days during the Test Fast (described above). You may have your blood drawn one additional time (3 times total) at baseline after your Test Fast before you start the study intervention. Your blood will be drawn again in the morning after an overnight fast at weeks 13, 26, and 52. Some subjects will also be asked to do a blood draw at week 78. We will use a sample of blood to determine your ABO and Rh blood type. In order to do this, a small portion of your blood sample will be mixed with monoclonal antibodies which will allow us to determine which proteins are on the surface of your red blood cells and which blood type you have.

j. Dietary Adherence, Effort, and Self-Efficacy Monthly Survey. You will be asked to rate on a 1-10 scale your answers to the following questions 1) how adherent you were to your prescribed study diet over the past week, 2) how hard was it for you to adhere to prescribed study diet over the past week, and 3) how likely is it that you feel you can adhere to your prescribed diet for the next month. We will also collect information on whether your home or work address has changed within this questionnaire. These measures are done at approximately weeks 4, 8, 13, 18, 22, 26, 30, 34, 39, 44, 48, and 52.

k. Medication Review. You will be asked to report any changes in your prescription and over the counter medications to study staff at baseline and at weeks 13, 26, 39, 52, and 78.

l. Stool Sample Collection: We will ask you to collect a stool sample at home using a kit that we provide. This is because recent research shows that what you eat impacts the bacteria that reside in your stomach and intestines (microbiome). Your microbiome may impact body weight. The stool collection is the same process used by hospitals around the world. We will provide a special paper that lies over the toilet, making the collection process very easy and clean. After the collection is done, the special paper is flushable. These measures are performed at baseline and at weeks 13, 26, and 52. Some subjects will be asked to perform this activity measurement at week 78.

m. 3 Minute Step Test: You will be asked to step on and off a 12 inch step 24 times per minute for 3 minutes to the beat of a metronome in order to assess your fitness level. We will measure your heart rate for 1 minute after this test. This measure is performed at baseline and at weeks 26, 52, and 78.

n. Assessment of Heart Rate Variability: You will be asked to wear a special monitor that records a continuous electrocardiogram (ECG) reading for approximately 30 minutes in order to measure the beat-to-beat variability of your heart rate. This is because we are interested in how stress impacts weight loss and the variability of your heart rate gives us an indication of the level of activity of your sympathetic nervous system which is how your body responds to stress. You will be asked to sit and stand for 1-2 minutes intervals during this test. This measure is performed at baseline and at weeks 26 and 52.

o. Highly Processed Food Withdrawal Scale: You will be asked to complete a brief (approximately 3 minute) online survey on study days 7,10,13, 16, 21 and 28 asking about symptoms that may occur when people begin a weight loss program and stop eating foods that have added levels of fat, refined carbohydrates (like sugar and flour), and/or salt.

p. Monthly Dietary Follow-up Questionnaires: You will be asked to complete a monthly online questionnaire about your efforts to continue losing weight or maintain your weight loss after the 12 month intervention ends. This questionnaire will be emailed to you at weeks 56, 60, 65, 70, 74, and 78.

**Note:** In the event of a public health emergency, natural disaster, severe weather, or for other compelling reasons at the discretion of the study PI, study group meetings and physical activity support sessions may held by Zoom videoconference or phone call and may not follow the exact timeline outlined in the protocol. Consent and screening procedures, including the medical history and physical exam with a study physician (with the exception of screening labs, ECG and vital signs) may be performed virtually (rather than in person) if needed for participant safety. Under these circumstances, study outcome measure visits may be delayed, postponed or cancelled as necessary for participant/staff safety and thus may not follow the exact timeline outlined in the consent. Under these extreme circumstances, study visits normally held at the AHWC and the outpatient CTRC may also be held at alternative locations at the University of Colorado Anschutz Medical Campus.

**Optional Consent for Additional Study Procedures**

a. Urine Collection to Evaluate Reproductive Hormones; Pre-menopausal women who have regular menstrual periods and are not using hormonal birth control will be asked to track the timing of menstrual cycles and collect daily urine samples to measure levels of reproductive hormones. This is because we are interested in understanding how obesity impacts reproductive hormones and cycle length, as well as if weight loss causes changes in reproductive hormones and/or cycle length. We will ask you to collect a morning urine sample at home using a container that we provide every day for a complete menstrual cycle. You will be asked to do this over one to two menstrual cycle(s) before you start the intervention (at baseline), over 3 menstrual cycles at the beginning of the study (around months 1-3), and over 1 menstrual cycle at the end of the study (around month 12). You will track menstrual cycle length with a calendar we provide during all 13-14 months. We will also measure testosterone and anti-Mullerian hormone in your baseline blood sample. Compensation will be provided for these measures.

Please indicate if you wish to participate in this optional study procedure of daily urine sample collection.

Yes  No  N/A _________Initials

b. Additional Questionnaires to Evaluate Trauma History: We are interested in learning more about your experiences during childhood and adolescence including history of physical or emotional trauma. Research has shown that experiences during development including history of trauma can influence health and health behaviors later in life. We will send you a secure link to complete a questionnaire from your home computer. This questionnaire takes about 10 minutes to complete and you need to complete it only once at baseline. Some of the items may be sensitive to some people or might make you feel uncomfortable or very rarely cause you to become upset about a traumatic experience you have had in the past. If any particular item makes you too uncomfortable, you do not have to answer. If any items on this questionnaire cause you distress, please contact study staff.

Please indicate if you wish to participate in this optional study procedure of completion of questionnaires to evaluate developmental and trauma history.

Yes  No _________Initials

**Optional Consent for Data and Specimen Banking for Future Research**

Dr. Catenacci would like to keep some of the data and blood samples that are taken during the study but are not used for other tests. If you agree, the data and samples will be kept and may be used in future research to learn more about the treatment of obesity. The research that is done with your data and samples is not designed to specifically help you. It might help people who have obesity and other diseases in the future. Reports about research done with your data and samples will not be given to you or your doctor. These reports will not be put in your health records. The research using your data and samples will not affect your care.

The choice to let Dr. Catenacci keep the data and samples for future research is up to you. No matter what you decide to do, it will not affect the care that you will receive as part of the study. If you decide now that your data and samples can be kept for research, you can change your mind at any time and contact your study doctor to let her know that you do not want Dr. Catenacci to use your data and samples any longer, and they will no longer be used for research. Otherwise, they may be kept until they are used up, or until Dr. Catenacci decides to destroy them.

When your data and samples are given to other researchers in the future, Dr. Catenacci will not give them your name, address, phone number or other information that will let the researchers know who you are.

Sometimes data and samples are used for genetic research (about diseases that are passed on in families). Even if your data and samples are used for this kind of research, the results will not be told to you and will not be put in your health records. Your data and samples will only be used for research and will not be sold. The research done with your data and samples may help to develop new products in the future, but there is no plan for you to be paid.

The possible benefits of research from your data and samples include learning more about what causes obesity and other diseases, how to prevent them and how to treat them. The greatest risk to you is the release of your private information. Dr. Catenacci will protect your records so that your name, date of birth, address and phone number will be kept private. The chance that this information will be given to someone else is very small. There will be no cost to you for any data or sample collection and storage by Dr. Catenacci.

Please read each sentence below and think about your choice. After reading each sentence, circle “yes” or “no.” If you have questions, please talk to your doctor or nurse. Remember, no matter what you decide to do about the storage and future use of your data and samples, you may still take part in the study.

1. I give my permissions for my data and blood to be kept by Dr. Catenacci for use in future research to learn more about how to prevent, detect, or treat obesity.

Yes  No _________Initials

2. I give my permissions for my data and blood to be used for research about other health problems (for example: causes of heart disease, osteoporosis, diabetes).

Yes  No _________Initials

3. I give my permission for my study doctor (or someone he or she chooses) to contact me in the future to ask me to take part in more research.

Yes  No _________Initials

You can cancel your permission to use your data and samples or to contact your for future research studies at any time by writing to the study’s Principal Investigator (PI), at the name and address listed below. If you do cancel your permission to use your data and/or blood samples, they will be disposed of and no longer used for research. If you withdraw consent to contact you for future studies, we will delete your from our recruitment list.

Victoria A. Catenacci, MD

University of Colorado Denver

Campus Box C263

12348 E. Montview Boulevard

Aurora, CO 80045

**Table 1 Study Procedures By Study Week**

|  | **Screening** | **Baseline** | **4** | **8** | **13** | **18** | **22** | **26** | **30** | **34** | **39** | **44** | **48** | **52** | **56** | **60** | **65** | **70** | **74** | **78** |
| --- | --- | --- | --- | --- | --- | --- | --- | --- | --- | --- | --- | --- | --- | --- | --- | --- | --- | --- | --- | --- |
| Study Visits | xx | xxxx | xx# |  | xx |  |  | xx |  |  | x |  |  | xx |  |  |  |  |  | x |
| Explanation of Study | x |  |  |  |  |  |  |  |  |  |  |  |  |  |  |  |  |  |  |  |
| Consent Form/HIPAA Authorization | x |  |  |  |  |  |  |  |  |  |  |  |  |  |  |  |  |  |  |  |
| Screening Questionnaires | x |  |  |  |  |  |  |  |  |  |  |  |  |  |  |  |  |  |  |  |
| Weight* | x | xxxx | xxxx | xxxx | xxxx | xx | xx | xx | xx | xx | xx | xx | xx | x |  |  |  |  |  | x |
| Height | x |  |  |  |  |  |  |  |  |  |  |  |  |  |  |  |  |  |  |  |
| Waist Circumference |  | x |  |  | x |  |  | x |  |  |  |  |  | x |  |  |  |  |  | x |
| Blood Pressure | x | x |  |  | x |  |  | x |  |  |  |  |  | x |  |  |  |  |  | x |
| EKG | x |  |  |  |  |  |  |  |  |  |  |  |  |  |  |  |  |  |  |  |
| Physical Exam and Medical History | x |  |  |  |  |  |  |  |  |  |  |  |  |  |  |  |  |  |  |  |
| Screening Labs (Blood Draw) | x |  |  |  |  |  |  |  |  |  |  |  |  |  |  |  |  |  |  |  |
| Urine Pregnancy Test (women only) | x | x |  |  | x |  |  | x |  |  |  |  |  | x |  |  |  |  |  | x |
| Test Fast | x |  |  |  |  |  |  |  |  |  |  |  |  |  |  |  |  |  |  |  |
| Group Meetings (weekly in months 0-3, every 2 weeks in months 4-12) |  | xxxx | xxxx | xxxx | xxxx | xx | xx | xx | xx | xx | xx | xx | xx | xx |  |  |  |  |  |  |
| DXA Body Composition |  | x | x# |  |  |  |  | x |  |  |  |  |  | x |  |  |  |  |  | x |
| Outcome Labs |  | xx(x#) |  |  | x |  |  | x |  |  |  |  |  | x |  |  |  |  |  | x |
| Stool collection |  | x |  |  | x |  |  | x |  |  |  |  |  | x |  |  |  |  |  | x |
| Daily urine collection# |  | x | x | x | x |  |  |  |  |  |  |  |  | x |  |  |  |  |  |  |
| DLW TDEE and EI |  | x | x# |  |  |  |  | x |  |  |  |  |  | x |  |  |  |  |  | x# |
| REE |  | xx | xx# |  |  |  |  | xx |  |  |  |  |  | xx |  |  |  |  |  | xx |
| 3 Minute Step Test |  | x |  |  |  |  |  | x |  |  |  |  |  | x |  |  |  |  |  | x |
| Heart Rate Variability Measure |  | x |  |  |  |  |  | x |  |  |  |  |  | x |  |  |  |  |  |  |
| 7 Day Diet and Exercise Record |  | x |  |  | x |  |  | x |  |  |  |  |  | x |  |  |  |  |  | x |
| ActivPAL Activity Monitor (7 days) |  | x |  |  | x |  |  | x |  |  |  |  |  | x |  |  |  |  |  | x |
| Dietary Adherence, Effort, and Self-Efficacy Survey |  | x | x | x | x | x | x | x | x | x | x | x | x | x |  |  |  |  |  |  |
| Monthly Dietary Follow-up Questionnaire |  |  |  |  |  |  |  |  |  |  |  |  |  |  | x | x | x | x | x | x |
| Outcome Questionnaires |  | x |  |  | x |  |  | x |  |  |  |  |  | x |  |  |  |  |  | x |
| Executive Function Measures |  | x |  |  | x |  |  | x |  |  |  |  |  | x |  |  |  |  |  | x |
| Concomitant Medication Review | x | x |  |  | x |  |  | x |  |  | x |  |  | x |  |  |  |  |  | x |
| Daily Home Weights |  | x | x | x | x | x | x | x | x | x | x | x | x | x | x | x | x | x | x | x |

# In subset of subjects only

*Outcome weights at baseline and week 4, 13, 26, 39, 52, and 78 are taken in the morning, wearing a hospital gown. In the IMF group, weight will be taken the morning following a fed day. Weight will also be measured weekly during months 0-3 and every other week during months 4-12 at the group-based behavioral weight loss sessions.

**Each “x” listed indicates the number of times the study procedure listed will occur at that time point. For example, the group meetings will occur 4 times between baseline and week 4.

**Generally, this will translate into the following number of visits:**

- **Screening:** 2 visits (1 x 2-3-hour visit, 1 x 30-minute visit)
- **Baseline:** 4-5 visits (one 6 hour visit followed by one 2 hour visit exactly 1 week apart and 2-3 x 30-minute visits)
- **Week 4:** 1 visit (30 minutes). In subset only; additional 2 visits (one 6 hour visit followed by one 2 hour visit exactly 1 week apart)
- **Week 13:** 2 visits (1 x 3 hour visit and 1 x 30-minute visit)
- **Week 26:** 3 visits (one 6 hour visit followed by one 2 hour visit exactly 1 week apart and 1 x 30-minute visit)
- **Week 39:** 1 visit (1 hour)
- **Week 52:** 3 visits (one 6 hour visit followed by one 2 hour visit exactly 1 week apart and 1 x 30-minute visit)
- **Week 78:** 1 visit (3 hours) In subset only; additional 2-3 visits (one 6 hour visit followed by one 2 hour visit exactly 1 week apart, and one 30 minute visit)

**This does not include measures you are asked to complete at home (such as stool and urine collection, weights, diet diaries, activity monitors, and questionnaires) or group meetings.**

**What are the possible discomforts or risks?**

**Risks of the Weight Loss Program:** You will be asked to decrease your calorie intake through daily caloric restriction (DCR) or intermittent fasting (IMF) to help you lose weight and maintain your weight loss.

*Common:* The most common side effects associated with dietary weight loss interventions (both DCR and IMF) are likely to be hunger, fatigue, trouble sleeping, irritability, anxiety, headaches, impaired concentration, and cold intolerance. It is possible you may miss school or work, have to take medications, or have to see a doctor if you experience one of these diet related conditions.

*Uncommon:* Occasionally people can experience constipation, nausea, abdominal discomfort, or diarrhea when changing their usual diet. It is possible you could develop weakness, dizziness, lightheadedness, tremor, psychological stress, or changes in your ability to think or process information when limiting your calorie intake. These conditions usually improve within a few weeks. Very rarely participation in a weight loss program can worsen an underlying eating disorder like anorexia, bulimia, or binge eating disorder or even cause someone to develop an eating disorder. Very rarely, participation in a weight loss program can cause ongoing stress in relationships with friends, family and co-workers. Very rarely, participation in a weight loss program can impact performance or impair ability to function at home, school, or at work.

*Rare and Serious:* The most serious but rare side effect of reducing your calorie intake is gallstone formation, which usually only occurs with extremely low-fat diets. Very rarely dehydration, kidney stones, hypoglycemia (low blood sugar), electrolyte abnormalities, confusion, changes in mood or behavior, changes in ECG, arrhythmias (irregular heart rhythms), syncope (passing out), or seizures can occur with fasting or significantly limiting energy intake.

**Risks of Exercise and 3 Minute Step Test:** You will be asked to increase your level of aerobic exercise to help you lose weight and maintain your weight loss. You will also be asked to perform a 3 minute step test to assess your fitness level at baseline, 26 weeks, 52 and 78 weeks.

*Common:* Aerobic exercise may cause sweating, fatigue, or feeling out of breath. These effects are normal, but the duration of exercise will gradually be increased to minimize these effects. It is also possible that you could develop soreness or injuries (most commonly in your feet, knees, legs, hips, or back) from exercising. If this occurs, you may need to see an outside medical doctor. If necessary, you will reduce the amount of exercise temporarily. It is possible you may miss school or work, have to take medications, or have to see a doctor if you experience one of these exercise related injuries or conditions.

*Uncommon*: Occasionally, aerobic exercise can cause headache, dizziness, shortness of breath, nausea, vomiting, high or low blood pressure, wheezing or lower extremity claudication. . Occasionally, people can trip or fall during aerobic exercise.

*Rare and Serious:* Aerobic exercise can rarely cause abnormal heartbeats (almost always reversible), chest pain, passing out, heart attack, stroke, or death. These are extremely rare events.

**Risk of Screening Medical History and Physical Exam:** In this study, you will be asked to complete a medical history interview and physical exam with the study physician, who will also review the results of the screening blood tests. It is possible that during these procedures we could find a medical condition or disease that you otherwise might not have known about. If this occurs, we will contact you and you may be referred to your primary care provider for follow up or to the emergency room if there is a serious abnormality.

**Risk of ECG and Assessment of Heart Rate Variability**: We will measure your heart rate and rhythm with an electrocardiogram (ECG) during the screening visit. We will measure your heart rate variability with a special monitor that records a continuous ECG reading for 15 minutes at the baseline, 26 week, and 52 weeks visits. There are no direct risks of performing an ECG. It is possible that the ECG might find an existing abnormal heart rhythm or condition that you otherwise might not have known about. If this occurs, we will contact you and you may be referred to your primary care provider for follow up or to the emergency room if there is a serious abnormality

**Risk of Screening and Outcome Questionnaires:** You may find completing the screening questionnaires and outcome questionnaires uncomfortable. You don’t have to answer any questions you do not want to answer. However, we need complete information about your baseline medical and social history, moods and eating attitudes to determine whether if it is safe for you to be in the study. Therefore, if you do not complete the **screening** questionnaires you will not be able to participate in the study. If questionnaires reveal you may be depressed, we will refer you to your primary care doctor for follow-up or to the emergency room if you are severely depressed. If questionnaires reveal you may have an eating disorder or a serious problem with alcohol or drug use, you will be withdrawn from the study and we will refer you to your primary care doctor for follow-up.

**Risk of Executive Function (Memory and Attention) Measures:** You may find completing the computer-based memory and attention tasks to be boring or uncomfortable. You don’t have to complete the memory and attention tasks if you do not want to. However, if you do not complete these measures you will not be paid for these measures.

**Risks of Having Blood Taken:** In this study we will need to get about 5 teaspoons of blood from you at each blood draw. Blood will be drawn once at screening and at weeks 13, 26 and 52, and twice at baseline. Some participants will have blood drawn at week 78. We will get blood by putting a needle into one of your veins and letting the blood flow into a glass tube. You may feel some pain when the needle goes into your vein. A day or two later, you may have a bruise where the needle went under the skin. Very rarely people may experience bleeding, infection, or numbness in the arm as a result of having blood drawn.

**Risks of the DXA:** As part of this study we will perform four to eight scans of your body. DXA is a way of looking inside the body by using X-rays. X-rays are a type of radiation. Your natural environment has some radiation in it. One DEXA scan will give you about the same amount of radiation that you would get from your environment in eight days.

**Risks of the Activity Monitor:** Rarely, skin irritation, redness, or rash can occur due to the activity monitor or the adhesives used to attach the activity monitor.

**Risks of Doubly Labeled Water:**  There are no known risks to consuming the stable isotopes in the doubly-labeled water used to measure TDEE. The labeled water tastes like regular tap water.

**Risks of Resting Energy Expenditure (REE):** You may feel claustrophobic during the measurement of REE. You will have the opportunity to try the hood prior to beginning the measurement.

**Risks of Stool, Urine, and Saliva Sample Collection.** There is minimal to no risk associated with the stool, urine, and saliva sample collection.

**Economic Risk:** It is possibly that you may need to pay for medical care as a result of participating in this study. For example, if the screening tests reveal an abnormality you may need to be further evaluated by an outside medical provider before you can participate in the study. If you experience an injury or illness during the study, you may need to be further evaluated by an outside medical provider.

**Risk of Loss of Confidentiality:** There is a risk that people outside of the research team will see your research information. We will do all that we can to protect your information, but it cannot be guaranteed.

**Risk if you Become Pregnant:** If you become pregnant during the study, the particular treatment or procedures involved in the study may involve risks to the embryo or fetus which are currently unclear.

There may be risks that are unknown at this time.

**What are the possible benefits of the study?**

This study is designed for the researcher to learn more about the best eating patterns for weight loss. This study is not designed to treat any illness or to improve your health. Also, there may be risks, as discussed in the section describing the discomforts or risks.

**Who is paying for this study?**

This research is being sponsored by the National Institutes of Health (NIH).

**Will I be paid for being in the study?**

You **will not be paid** for completion of screening procedures or baseline measures.

You will be paid for completion of other study outcome measures as follows:

.

- **Week 4 Measures:** You will be paid a total of **$20-$100** if you complete all outcome measures at week 4. Payment will be pro-rated as follows: You will be paid $20 for anthropometric measures (body weight). You will be paid $60 if you complete both visits for the doubly-labeled water measure of TDEE at week 4 (including the REE measures) and $20 for the DXA body composition assessment at week 4. **NOTE: only a subset of subjects will be asked to perform the doubly-labeled water measure of TDEE and DXA body composition assessment at week 4.**
- **Week 13 Measures:** You will be paid a total of **$140** if you complete all outcome measures at week 13. Payment will be pro-rated as follows: $20 for anthropometric measures (body weight, blood pressure, and waist circumference), $20 for a complete 7 day diet and exercise record, $20 for the blood draw, $20 for a complete 7-day activity monitor assessment,, $30 for completion of questionnaires, $10 for completion of executive function measures, and $20 for a stool collection. **NOTE: only a subset of subjects will be asked to perform the activity monitor measures.**
- **Week 26 Measures:** You will be paid a total of **$220** if you complete all outcome measures at week 26. Payment will be pro-rated as follows: $20 for anthropometric measures (body weight, blood pressure, waist circumference, 3 minute step test and heart rate variability measure), $20 for completion of 1-2 DXA body composition assessments, $60 for completion of both visits for the DLW assessment of TDEE (including the REE measures), $20 for complete 7-day activity monitor assessment, $20 for a complete 7-day diet and exercise record, $20 for the blood draw, $30 for completion of questionnaires, $10 for completion of executive function measures, and $20 for a stool collection.
- **Week 39 Measures**: You will be paid a total of **$20** if you complete all outcome measures at week 39. Payment will be pro-rated as follows: you will receive $20 for completing a body weight.
- **Week 52 Measures:** You will be paid a total of **$220** if you complete all outcome measures at week 52. Payment will be pro-rated as follows: $20 for anthropometric measures (body weight, blood pressure, waist circumference, 3 minute step test, and heart rate variability measure), $20 for completion of 1-2 DXA body composition assessments, $60 for completion of both visits for the DLW assessment of TDEE including the REE measures, $20 for complete 7-day activity monitor assessment, $20 for a complete 7-day diet and exercise record, $20 for the blood draw, $30 for completion of questionnaires, $10 for completion of executive function measures, and $20 for a stool collection.
- **Week 78 Measures:** You will be paid a total of **$100-220** if you complete all outcome measures at week 78. Payment will be pro-rated as follows: $20 for body weight and waist circumference, $20 for DXA body composition assessment, $20 for a complete 7-day diet and exercise record, $20 for complete 7-day activity monitor assessment, $40 for completion of both visits for the DLW assessment of TDEE, $20 for assessment of REE, $20 for the blood draw, $20 for a stool collection, $30 for completion of questionnaires, and $10 for completion of executive function measures. **NOTE: only a subset of subjects will be asked to perform the activity monitor, DLW assessment of TDEE, blood draw, and/or REE assessments at week 78.**

Thus, you will receive a total of **$720-920** if you complete all of the study assessments. You could receive an additional $10 for the PBRC COVID Survey if you are asked to complete this.

- **Additional Optional Assessments**

You will be paid a total of **$375-$445** if you participate in the collection of daily urine for assessment of reproductive hormones. Payment will be pro-rated as follows: you will receive $70 per full month of urine collected plus $25 for tracking and reporting your menstrual cycle length for all 13 months. **NOTE: only a subset of pre-menopausal women with normal menstrual cycles and not on hormonal birth control will be asked to participate in these optional procedures.**

Occasionally, an assessment may need to be repeated to obtain an adequate measurement. For example, if the physical activity monitor did not work properly during the data collection period or if the body position on the DXA scan was not optimal. If this occurs, we will ask you to repeat the outcome measure that was not adequate. However, you will only be compensated for one outcome measure.

You will be weighed at each weight loss group meeting and you will be asked to keep weekly food logs to review with the group leader but you will not be paid for these measures.

The study will also pay for a 1 year membership to the AHWC Fitness Center during the 12 month intervention phase of the study on a monthly pro-rated basis. This is being done to provide you a place to exercise and as additional compensation for your time in attending group meetings and performing outcome measures. You must complete the monthly dietary adherence surveys, attend at least 50% of group weight loss classes, and complete study outcome measures to continue to receive this benefit. We will evaluate your study standing every few months to determine if you will continue to receive this benefit. Payment of the AHWC Fitness Center membership fee is not transferrable to another individual. Payment of the AHWC Fitness Center membership fee cannot be converted to cash compensation nor can it be refunded or credited to you if you do not use the facility. AHWC Fitness Center memberships may be extended for longer than 12 months in the event of extended fitness center closures due to public health emergency.

If you complete all 6 times points of the Highly Processed Food Withdrawal Scale on study days 7,10,13,16, 21 and 28 you will be entered into a drawing for a gift certificate for a 50 minute massage at the AHWC. One winner will be selected form each study cohort.

If you complete all 5 of the Monthly Dietary Follow-up Questionnaires during study weeks 56, 60, 65, 70, and 74 you will be entered into a drawing for a $100 gift card. One winner will be selected from each study cohort.

If you leave the study early, or if we have to take you out of the study, you will be paid only for the assessments you have completed. If you leave the study early, or if we have to take you out of the study, we will no longer continue to pay your AHWC Fitness Center monthly membership fee.

It is important to know that payment for participation in a study is taxable income.

**Will I have to pay for anything?**

You may need to pay for parking during study visits and group weight loss meetings.

Parking in the visitor lots near the AHWC is $1 per hour on weekdays, and a flat rate of $1 on weekends and after 4 PM on weekdays.

You may need to purchase appropriate exercise footwear (properly fitting and supportive sneakers) to wear during your exercise. Appropriate footwear is important in preventing exercise-related injuries such as ankle sprains and shin splints.

You may need to pay for medical care to follow up abnormalities that may be found during screening procedures or outcome measures. You may need to pay for medical care for any injuries or illness that occurs during or as a result of your participation in the study.

**Is my participation voluntary?**

Taking part in this study is voluntary. You have the right to choose not to take part in

this study. If you choose to take part, you have the right to stop at any time. If you refuse or decide to withdraw later, you will not lose any benefits or rights to which you are entitled.

If you do withdraw from the study, we will ask you to come to the clinic to get a final weight and return all study materials that you have in your possession. Once you return all study materials, you will be compensated $25 for your final weight measurement.

**Can I be removed from this study?**

You may be taken out of this study if the study doctor thinks it is not safe for you to be in the study. You will be removed from the study if you become pregnant or develop a medical condition that makes you ineligible for the study. You will be removed from the study if you choose to participate in another weight or exercise program or research study. You will be removed from the study if you chose to take a weight loss medication or supplement. You may be removed from the study if you do not regularly attend the group weight loss meetings. You may be removed from the study if you do not regularly complete study outcome measures. You may be removed from the study if you are unwilling to follow the diet condition you are assigned to receive. You can be taken out of the study even if you do not want to leave the study. Also, the sponsor may stop the study at any time.

**Certificate of Confidentiality**

This study has been issued a Certificate of Confidentiality from the federal government to help protect your privacy. The Certificate prohibits the researchers from disclosing your name, or any identifiable information, document or biospecimen from the research, with the exceptions listed below. A certificate provides protections against disclosing research information in federal, state, or local civil, criminal, administrative, legislative or other proceedings.

These protections apply only to your research records. The protections do not apply to your medical records.

The researchers may disclose your name or identifiable information, document or biospecimen, under the following circumstances:

- To those connected with the research,
- If required by Federal, State or local laws,
- If necessary for your medical treatment, with your consent,
- For other scientific research conducted in compliance with Federal regulations,
- To comply with mandated reporting, such as a possible threat to harm yourself or others, reports of child abuse, and required communicable disease reporting, or
- Under other circumstances with your consent.

A Certificate of Confidentiality does not protect information you or a member of your family voluntarily release.

**What happens if I am injured or hurt during the study?**

If you have an injury while you are in this study, you should call Dr. Victoria A. Catenacci immediately. Her phone number is (303) 724-9052. We will arrange to get you medical care if you have an injury that is caused by this research. However, you or your insurance company will have to pay for that care.

**Who do I call if I have questions?**

The researcher carrying out this study is Victoria A. Catenacci, MD. You may ask any questions you have now. If you have questions, concerns, or complaints later, you may call Dr. Catenacci at (303) 724-9052. You will be given a copy of this form to keep. The main person to call if you have questions about this study is Dr. Catenacci. You can also talk to a Subject Advocate at the Clinical Translation Research Center (CTRC). The phone number there is (720) 848-6662.

You may have questions about your rights as someone in this study. You can call Dr.

Catenacci with questions. You can also call the responsible Institutional Review

Board (COMIRB). You can call them at (303) 724-1055.

Information about this study can also be found on clinicaltrials.gov.

**Who will see my research information?**

The University of Colorado Denver (UCD) and its affiliated hospital(s) have rules to protect information about you. Federal and state laws including the Health Insurance Portability and Accountability Act (HIPAA) also protect your privacy. This part of the consent form tells you what information about you may be collected in this study and who might see or use it.

The institutions involved in this study include:

- University of Colorado Denver
- University of Colorado Hospital

We cannot do this study without your permission to see, use and give out your information. You do not have to give us this permission. If you do not, then you may not join this study.

We will see, use and disclose your information only as described in this form and in our Notice of Privacy Practices; however, people outside the UCD and its affiliate hospitals may not be covered by this obligation.

We will do everything we can to maintain the confidentiality of your personal information but confidentiality cannot be guaranteed.

The use and disclosure of your information has no time limit. You can cancel your permission to use and disclose your information at any time by writing to the study’s Principal Investigator (PI), at the name and address listed below. If you do cancel your permission to use and disclose your information, your part in this study will end and no further information about you will be collected. Your cancellation would not affect information already collected in this study.

Victoria A. Catenacci, MD

University of Colorado Denver

Campus Box C263

12348 E. Montview Boulevard

Aurora, CO 80045

Both the research records that identify you and the consent form signed by you may be looked at by others who have a legal right to see that information, such as:

- Federal offices such as the Food and Drug Administration (FDA) and the Office of Human Research Protections (OHRP) that protect research subjects like you.
- People at the Colorado Multiple Institutional Review Board (COMIRB)
- The study doctor and the rest of the study team.
- The National Institute of Health (NIH), who is the institution paying for this research study.
- Officials at the institution where the research is conducted and officials at other institutions involved in this study who are in charge of making sure that we follow all of the rules for research.
- Genetic data will be registered with the database Genotypes and Phenotypes (dBGaP)

We might talk about this research study at meetings. We might also print the results of this research study in relevant journals. But we will always keep the names of the research subjects, like you, private.

You have the right to request access to your personal health information from the Investigator. To ensure proper evaluation of test results, your access to these study results may not be allowed until after the study is completed.

**The investigator (or staff acting on behalf of the investigator) will use your information for the research outlined in this consent form. They will also make some of the following health information about you collected in this study available to:** Biomarker Testing Services.

**Information about you that will be seen, collected, used and disclosed in this study:**

- Name and Demographic Information (age, sex, ethnicity, address, phone number, etc.
- Your social security number
- Portions of your previous and current Medical Records that are relevant to this study, including but not limited to Diagnosis(es), History and Physical, laboratory or tissue studies, radiology studies, procedure results
- Research Visit and Research Test records
- Psychological and mental health tests
- Alcoholism, Alcohol or Drug abuse
- Tissue samples and the data with the samples.

**What happens to Data and Blood that are collected in this study?**

Scientists at the University of Colorado Denver and the hospitals involved in this study work to find the causes and cures of disease. The data and blood collected from you during this study are important to this study and to future research. If you join this study:

- The data and blood given by you to the investigators for this research no longer belong to you.
- Both the investigators and any sponsor of this research may study your data and blood collected from you.
- If data or blood are in a form that identifies you, UCD or the hospitals involved in this study may use them for future research only with your consent or Institutional Review Board (IRB) approval.
- Any product or idea created by the researchers working on this study will not belong to you.
- There is no plan for you to receive any financial benefit from the creation, use or sale of such a product or idea.

**HIPAA Authorization for Optional Additional Study Procedures**

In this form, you were given the option to agree to additional, optional research procedures. You must also give us your permission, under HIPAA rules, to use and disclose the information collected from these optional procedures, as described above.

If you decline to give us permission to use and disclose your information, you cannot take part in these optional procedures, but you can still participate in the main study. Please initial next to your choice:

_____ I give permission for my information, from the optional procedures I have agreed to above, to be used and disclosed as described in this section.

_____ I **do not** give permission for my information for any optional procedures to be used and disclosed; I understand that I will not participate in any optional procedures.

**Agreement to be in this study and use my data**

I have read this paper about the study or it was read to me. I understand the possible risks and benefits of this study. I understand and authorize the access, use and disclosure of my information as stated in this form. I know that being in this study is voluntary. I choose to be in this study: I will get a signed and dated copy of this consent form.

Signature: Date:

Print Name:

Consent form explained by: Date:

Print Name:
